# Integrative Analysis of the Wheat *PHT1* Gene Family Reveals A Novel Member Involved in Arbuscular Mycorrhizal Phosphate Transport and Immunity

**DOI:** 10.3390/cells8050490

**Published:** 2019-05-22

**Authors:** Yi Zhang, Lizong Hu, Deshui Yu, Kedong Xu, Ju Zhang, Xiaoli Li, Pengfei Wang, Guo Chen, Zhihui Liu, Chunfeng Peng, Chengwei Li, Tiancai Guo

**Affiliations:** 1 The Collaborative Innovation Center of Henan Food Crops, Agronomy College, Henan Agricultural University, Zhengzhou 450002, China; yizhang0401@sina.com (Y.Z.); wangpf2013@163.com (P.W.); 2 Key Laboratory of Plant Genetics and Molecular Breeding, Zhoukou Normal University, Zhoukou 466001, China; hulizong@126.com (L.H.); ds-yu@163.com (D.Y.); xukd1107@126.com (K.X.); zjtt603@126.com (J.Z.); xiaoli890107@163.com (X.L.); cgzknu@sina.com (G.C.); lzhzknu@sina.com (Z.L.); pcfzknu@sina.com (C.P.); 3 Henan Key Laboratory of Crop Molecular Breeding & Bioreactor, Zhoukou Normal University, Zhoukou 466001, China; 4 Henan Engineering Research Center of Grain Crop Genome Editing, Henan Institute of Science and Technology, Xinxiang 453003, China

**Keywords:** arbuscular mycorrhizal, *PHT1* gene family, expression pattern, subcellular localization, mycorrhizal-specific/inducible gene, virus-induced gene silencing, immunity

## Abstract

Phosphorus (P) deficiency is one of the main growth-limiting factors for plants. However, arbuscular mycorrhizal (AM) symbiosis can significantly promote P uptake. Generally, PHT1 transporters play key roles in plants’ P uptake, and thus, *PHT1* genes have been investigated in some plants, but the regulation and functions of these genes in wheat (*TaPHT1*) during AM symbiosis have not been studied in depth. Therefore, a comprehensive analysis of *TaPHT1* genes was performed, including sequence, phylogeny, *cis*-elements, expression, subcellular localization and functions, to elucidate their roles in AM-associated phosphate transport and immunity. In total, 35 *TaPHT1s* were identified in the latest high-quality bread wheat genome, 34 of which were unevenly distributed on 13 chromosomes, and divided into five groups. Sequence analysis indicated that there are 11 types of motif architectures and five types of exon-intron structures in the *TaPHT1* family. Duplication mode analysis indicated that the *TaPHT1* family has expanded mainly through segmental and tandem duplication events, and that all duplicated gene pairs have been under purifying selection. Transcription analysis of the 35 *TaPHT1s* revealed that not only known the mycorrhizal-specific genes *TaPht-myc*, *TaPT15-4B* (*TaPT11*) and *TaPT19-4D* (*TaPT10*), but also four novel mycorrhizal-specific/inducible genes (*TaPT3-2D*, *TaPT11-4A*, *TaPT29-6A*, and *TaPT31-7A*) are highly up-regulated in AM wheat roots. Furthermore, the mycorrhizal-specific/inducible genes are significantly induced in wheat roots at different stages of infection by colonizing fungi. Transient *Agrobacterium tumefaciens*-mediated transformation expression in onion epidermal cells showed that TaPT29-6A is a membrane-localized protein. In contrast to other AM-specific/inducible *PHT1* genes, *TaPT29-6A* is apparently required for the symbiotic and direct Pi pathway. *TaPT29-6A*-silenced lines exhibited reduced levels of AM fungal colonization and arbuscules, but increased susceptibility to biotrophic, hemi-biotrophic and necrotrophic pathogens. In conclusion, *TaPT29-6A* was not only essential for the AM symbiosis, but also played vital roles in immunity.

## 1. Introduction

Phosphorus (P) is an essential macronutrient for plants, with diverse structural roles as a constituent of various cellular macromolecules, and physiological roles as a participant in energy metabolism, signal transduction, photosynthesis and numerous other metabolic processes [1,2]. Despite its abundance in the environment (the 11th most abundant element) [3], phosphorus is one of the least available plant nutrients globally, as it mostly exists in soil in complex, insoluble and organic forms that plants cannot directly acquire [1]. Plants’ primary source of P in soil is inorganic phosphate (Pi) [2], and plants can reportedly acquire at most 30% of applied Pi [4], which has led to excess application of Pi fertilizers, water eutrophication and soil hardening [5]. Thus, to protect water and soil resources, and improve crop production, there is an urgent need to identify genes and elucidate mechanisms associated with phosphate acquisition and utilization [4]. To facilitate such efforts we need to elucidate plants’ responses to low-phosphate stress and their evolution of strategies that enhance P acquisition and utilization. These include alterations in root morphology, modification of the soil chemistry around roots (e.g., by reduction of pH or exudation of organic anions), production and secretion of organic acids and phosphatases, formation of symbiotic associations with arbuscular mycorrhizal (AM) fungi and activation of high-affinity Pi transporters [4].

Increasing studies of molecular biology and functional genomics of model plants have provided abundant knowledge of Pi transport mechanisms recently, including identification of various phosphate (Pi) transporters with different affinities [6,7,8,9]. According to their sequences, structures, locations and functions, these transporters have been classified into four families, named PHT1, PHT2, PHT3 and PHT4 [6,7]. PHT1 Pi transporters play the main role in Pi uptake from soil by plant roots. All members of the family are H^+^/Pi symporters, with high Pi affinity, and strongly expressed in roots [10,11], especially rhizodermal cells and the outer cortex, and most of them are up-regulated in Pi-deprived plants [12], The PHT1 family is also the most widely studied Pi transporter family in plants. The first plant *PHT1* gene to be cloned was from *Arabidopsis* [13] and exhibits similarities to genes encoding Pi transporters in *Saccharomyces cerevisiae* (*PHO84*) [14], *Neurospora crassa* (*PHO5*) [15], and *Glomus versiforme* (*GvPT*) [16]. In the model plants *Arabidopsis* and rice, nine and 13 *PHT1*s, respectively, have been identified and intensively studied [12,17].

AM are arguably the most common underground symbionts, establishing beneficial symbiosis with approximately 80% of terrestrial plant species [18]. They are obligate symbionts and depend entirely on the host plant to provide them with carbon. In exchange, they assist the plant in acquiring nutrients, especially Pi. It was recently speculated that in AM symbiosis plants may receive virtually all of their phosphorus via their fungal symbiont [19,20,21]. The far-reaching extraradical mycelium of AM grows beyond the phosphate depletion zone resulting from high plant phosphate uptake and low phosphate diffusion rates in soil. It may extend 100 times further than plant root hairs, it is highly branched and may provide potent functional extensions of the plant root system [1,17]. AM hyphae also participate in mineralization of organic P in soil, and establishment of AM association can induce expression and secretion of plant-derived acid phosphatases in the rhizosphere, thereby liberating further Pi [4].

Most *PHT1*s are reportedly up-regulated in Pi-deprived plants [12] and two classes have known involvement in Pi transport in AM symbiosis: mycorrhiza-specific Pi transporters and mycorrhiza-inducible Pi transporters [22,23]. Mycorrhiza-inducible Pi transporters (such as *StPT3* in potato [24], *HvPt8* in barley [25], *OsPT13* in rice [26], *SmPT3* in eggplant [27], *CfPT3* in pepper [27], and *NtPT3* in tobacco [27]) are expressed in non-symbiotic roots, but more strongly in symbiotic roots. Mycorrhiza-specific Pi transporters (such as *OsPT11* in rice [17], *MtPT4* in alfalfa [28], *SlPT4* in tomato [29], *TaPht-myc* in wheat [25], and *PtPT10* in *Populus trichocarpa* [30]) are only expressed in AM-colonized plants, but their functions have not been thoroughly characterized. *MtPT4* and *OsPT11*, the first mycorrhiza-specific Pi transporter genes identified in dicot and monocot plants, respectively, have been characterized in the most detail, and shown to be essential for the plants’ AM-mediated Pi transport [17,22]. Mutation of *MtPT4* and *OsPT11* significantly affects formation of arbuscules, the main sites of signal and nutrient exchange, and overall fungal colonization [22,31]. Additionally, promoters of the two genes are exclusively expressed in arbusculated cells [22,26]. In contrast, the tomato homolog of *OsPT11*, *SlPT4*, is not essential for establishment of AM symbiosis, indicating considerable genetic redundancy in tomato, in which AM symbiosis also triggers expression of two other *PHT1*s: *SlPT3* and *SlPT5* [29]. These findings prompted speculation that *OsPT11*-like proteins may be supplementary Pi transporters in some plants, with functions that have not yet been determined.

In wheat (*Triticum aestivum*), one of the most important food crops, AM symbiosis improves Pi use efficiency, and thus plays a major role in globally sustainable agricultural and food security [32]. *TaPht-myc*, the first mycorrhizal-specific *PHT1* in wheat [25], as well as *TaPT10*, *TaPT11* and *TaPT12*, are reportedly induced in roots colonized by AM [33], but their functions have not been clearly elucidated. Potential roles of a few wheat PHT1 members in phosphorus metabolism have been identified [34,35,36], but functional verification and far more detailed general data are needed to elucidate the roles of Pi transporters in AM symbiosis. Thus, the aims of this study were to: identify all *PHT1*s in wheat (*TaPHT1*s) by comprehensive genome-wide analysis; analyze their phylogenetic evolution, gene structure, chromosomal organization, and expression patterns in response to various AM fungi (under high and low Pi conditions); and verify functions of *TaPHT1* in phosphate transport, AM symbiosis and immunity. The results should strengthen our understanding of the evolutionary expansion, conservation and functional divergence of *PHT1*s in wheat, and more generally roles of *PHT1*s in these processes.

## 2. Materials and Methods

### 2.1. Retrieval of PHT1 Genes and Sequence Analysis

Genomic sequences of wheat were retrieved from the International Wheat Genome Sequencing Consortium (http://www.wheatgenome.org/), and data on other plants were obtained from the TAIR (http://www.arabidopsis.org/), RAP-DB (https://rapdb.dna.affrc.go.jp/), Phytozome (https://phytozome.jgi.doe.gov/pz/portal.html), SDG (http://www.yeastgenome.org/) and NCBI (https://www.ncbi.nlm.nih.gov/) databases. Three methods were used to identify putative *PHT1s* in wheat and other plants. First, the NCBI database was searched using relevant keywords. Second, sequences of well-known rice and *Arabidopsis* PHT1 proteins [6,12] were used in BLASTP searches of the mentioned protein databases, with an E-value cutoff of 1e-5. Third, a Hidden Markov Model (HMM) of 58 amino acids containing the core motif “GGDYPLSATIxSE” and its flanking sequences was used by HMMER to identify candidate *PHT1* genes with default parameters. Subsequently, all redundant sequences were manually removed and the remaining PHT1 sequences were further verified by Pfam (http://pfam.sanger.ac.uk/).

The PHT1 protein sequences were aligned by Clustal X (Version 1.83, Belfield, Dublin, Ireland; http://www.clustal.org/) with default parameters. Then, the maximum likelihood (ML) method implemented in MEGA (Version 7.0, Tempe, AZ, USA; https://www.megasoftware.net/) was used to build a phylogenetic tree with bootstrap values generated from 1000 replicates. This tree, with conserved Sugar_tr domains (PF00083) of PHT1 proteins, was annotated and visualized by iTOL (Version 3.0, University of Würzburg, Würzburg, Germany; https://itol.embl.de/). The conserved motifs in PHT1 proteins were investigated using the online MEME (Version 5.0.4, University of Washington, Seattle, WA, USA; http://meme-suite.org/) package, with the following parameters: number of repetitions, any; maximum number of motifs, 20; and optimum motif width, 12-200 residues. Motif 2, shared by almost all the PHT1 proteins was displayed using Weblogo (Version 2.8.2, University of California, Berkeley, CA, USA; http://weblogo.berkeley.edu/logo.cgi). Moreover, exon/intron architectures of *PHT1*s in rice, *Arabidopsis* and wheat were analyzed by GSDS (Version 2.0, Peking University, Beijing, China; http://gsds.cbi.pku.edu.cn/).

Identified *TaPHT1*s were localized on wheat chromosomes, according to the wheat GFF3 file from IWGAC (IWGSC RefSeq Version 1.0, URGI, Versailles, France; https://www.wheatgenome.org/), their duplication patterns were investigated using MCScanX [37], and the wheat proteome was aligned by local BLAST (Version 2.2.17, NCBI, Bethesda, MD, USA; https://blast.ncbi.nlm.nih.gov/Blast.cgi) searches. Positions of *TaPHT1* genes and their synteny relationships were further visualized by Circos [38]. Each pair of duplicated genes was submitted to the KaKs_Calculator for calculation of non-synonymous (Ka) to synonymous (Ks) substitution ratios [39]. The duplication time (T) was calculated using the formula T = Ks/2λ for grasses, with λ = 6.5 × 10^−9^ [40]. Putative trans-membrane segments of TaPHT1 proteins were predicted by TMHMM (Version 2.0, CBS, Copenhagen, Denmark; http://www.cbs.dtu.dk/services/TMHMM/). The 2-kb upstream regions of the 35 identified *TaPHT1*s were downloaded as putative promoter regions, and corresponding *cis*-regulatory elements were analyzed using the PLACE database with manual adjustment [41]. 

### 2.2. Plant Materials and Fungus Treatments

Seeds of Chinese winter wheat cultivar Zhoumai 26 were surface-sterilized twice with 2.5% sodium hypochlorite solution for 8 min, then washed extensively with sterile water, and vernalized for 7 days at 4 °C in the dark. The aseptic plantlets were then transferred to pots, containing a sterilized soil: river sand mixture (1:2, *v*/*v*). Sand-based autoclaved inocula containing 60 g *Funneliformis mosseae* (Nicol. & Gerd, BGC XZ02A, isolated from the rhizosphere in Dangxiong, Tibet, China) (formerly known as *Glomus mosseae*) or *G. versiforme* (BGC NM04B) were each used to inoculate 20 plantlets. For non-mycorrhizal plants, the same amount of autoclaved inoculum was added to the mixture. Plants were grown in a greenhouse providing 20 °C, 12-h day/12 °C 12-h night cycles at 60% relative humidity. All the inoculated and control plants were watered with half-strength Hoagland’s nutrient solution twice a week, supplemented with 5 or 500 µM KH_2_PO_4_, respectively providing insufficient (‘low’) P and sufficient (‘high’) P nutrient solutions [42]. There were three replicates for each treatment. A total of 360 plants were harvested 6 weeks after AM inoculation, then their biomass and Pi concentrations of shoots and roots of 160 AM inoculated and 80 uninoculated plants were measured, and the remaining 120 plants were used to analyze the mycorrhizal infection. 

*Bipolaris sorokiniana*, a hemibiotrophic fungus (which causes root rot), isolated from wheat and provided by Henan Agriculture University, was cultured at 20 °C on potato dextrose agar (PDA). After 15 days, spores were harvested in sterile water, and the resulting suspension was diluted to approximately 4 × 10^4^ spores per mL. For pathogen inoculation, 10-day-old seedlings were inoculated with the spore suspension near their stem bases, following a previously described method, then root tissues were collected at 2, 3, 4, 5, and 6 days post-inoculation (dpi) to analyze *PHT1* expression patterns [43]. *Gaeumannomyces graminis* var. *tritici* (*Ggt*), a necrotrophic pathogen, kindly provided by Henan Academy of Agricultural Science, was used to inoculate another set of seedlings, again following a previously published method [44]. The two pathogens infected plants were cultured in a growth chamber providing a 22 °C, 16 h light/15 °C, 8 h dark regime at 70% relative humidity. *TaPR4A/B* (pathogenesis-related type 4) were previously shown to be strongly induced by several pathogenic fungus [45,46], and thus were used as markers for pathogenicity. Roots were collected at 2, 3, 4, 5 and 6 dpi for RNA extraction. The naturally occurring biotrophic fungus *Blumeria graminis* f.sp. *tritici* (*Bgt*), isolated from a field in Zhoukou, Henan province, China (33°62ʹN, 114°65ʹE), was maintained on wheat cv. Aikang 58 plants, kept under a 22 °C, 16-h light/20 °C, 8-h dark regime with 60% air humidity.

### 2.3. RNA Extraction and Quantitative Real-Time PCR

Total RNA was isolated from samples described previously using the guanidine thiocyanate extraction method with Trizol reagent (Life Technologies, Grand Island, NY, USA). After extraction, the RNA samples were treated with DNase I (TaKaRa Bio Inc., Shiga, Japan) to eliminate trace contaminants of genomic DNA. cDNA was synthesized using a PrimeScript^RT^ Perfect Real Time reagent kit (TaKaRa). The synthesized cDNAs were used as templates in the following PCR reactions. Relative expression levels of *TaPHT1* in roots and leaves of wheat plants exposed to the AM used in the tests in both high and low P conditions were assayed by real-time RT-PCR analysis. For this, a CFX96™ Real-Time PCR Detection System (Bio-Rad, Hercules, CA, USA) with SYBR^®^ Premix Ex Taq™ (Tli RNaseH Plus, Takara Bio Inc.) was used, with amplification settings of 95 °C for 3 min, followed by 40 cycles of 95 °C for 10 s, and finally 60 °C for 30 s. All assays for a particular gene were performed three times synchronously under identical conditions, and RNA transcript fold changes were calculated using the 2^−ΔΔCt^ method [47]. Relative levels of transcripts of each wheat *PHT1* were standardized to the expression level of the constitutively expressed wheat *Actin* gene (KC775780). The specificity of primer sets designed for the qRT-PCR analysis (Table S4) was confirmed by sequencing after the PCR reaction. Three biological replicates were used for all these analyses.

### 2.4. Subcellular Location of TaPT29-6A in Living Onion

For subcellular localization assays, entire coding sequences of *TaPT29-6A* with no stop codon were amplified using gene-specific primers (Table S4). *TaPT29-6A* was fused in-frame to the 5′ terminus of the green fluorescent protein (*GFP*) gene in the pSAT6-GFP vector after digestion with EcoRI, and SalI. The recombinant vectors and pSAT6-GFP plasmid were transformed separately into *A. tumefaciens* strain GV3101 for further transient transformation in living onion epidermal cells, as previously described [48]. GFP fluorescence signals were measured with microruler under a microscope (Olympus, Tokyo, Japan). Images of epidermal cells emitting GFP signals were taken using a motorized fluorescence microscope equipped with a mirror unit (U-MSWB2), dichroic mirror (DM500), excitation filter (BP470-490) and barrier filter (BA520IP).

### 2.5. TRV-Mediated Silencing of Phosphate Transporter Gene in Wheat

To further verify functions of *TaPT29-6A* in AM-mediated and direct Pi transport, the gene was silenced using the tobacco rattle virus (TRV)-mediated virus-induced gene silencing (VIGS) (TRV-VIGS) system. Sequence alignment revealed that *TaPT29-6A*, *TaPT30-6D* and *TaPht-myc* are homologous alleles, and highly conserved 183-bp nucleotide segments were selected as VIGS targets (Figure S1). The VIGS sequences of *TaPT29-6A* was amplified from Zhoumai 26, and In-Fusion HD Cloning Plus (Takara Bio Inc. [49] was used to transform TRV with pYL156 plasmids carrying *TaPT29-6A* (generating TRV:*TaPT29-6A*). The empty pYL156 vector was also transformed into TRV as a negative control, designated TRV:00. Seeds of Zhoumai 26 were used to generate silenced seedlings, following the whole-plant silencing method with minor modification [50], and non-silenced controls (containing TRV:00). Sixteen days after virus inoculation, total RNA was extracted from roots and leaves of silenced and non-silenced plants to quantify *TaPT29-6A* expression levels, following procedures described in “RNA Extraction and Quantitative Real-time PCR”.

### 2.6. Determination of Phosphorus Contents

To measure their total Pi concentrations, plant samples were dried at 105 °C for 2–3 h and 80 °C for 30 min. Then 0.2 g portions of dried crushed plant sample powder were digested in glass tubes containing 5 mL concentrated H_2_SO_4_ and 3 mL of 30% hydrogen peroxide for 24 h. The tubes were heated to 180 °C for 20 min, then to 280 °C for 10 min. Next, 50 μL hydrogen peroxide was added every 10 min, until the solution turned colorless [51]. After cooling, each predigested sample was diluted to 100 mL with deionized water. The total Pi concentration in the solution was determined using the molybdate-blue colorimetric method, and a TU-1900 UV-visible spectrophotometer (Beijing Purkinje General Instrument, Beijing, China) to read absorbance at 700 nm [52]. 

### 2.7. Functional Verification of TaPT29-6A-Silenced Plants’ Response to Representative Fungal Colonists

To validate the function of *TaPT29-6A* in the responses of wheat to representatives of several types of fungal colonists, sets of 30 silenced wheat seedlings were inoculated with either the AM *F. mosseae*, or hemi-biotrophic fungus *B. sorokiniana*, or necrotrophic pathogen *G. graminis* var. *tritici* (*Ggt*), and or biotrophic pathogen *B. graminis* f. sp. *tritici* (*Bgt*). The *TaPT29-6A*-silenced and TRV:00 control plants were inoculated with *F. mosseae*, and the mycorrhizal colonized roots were harvested six weeks later. Fresh collected AM-colonized wheat roots were stained with WGA-AlexaFluor 488 (Life Technologies, Carlsbad, CA, USA), as previously described [53]. Percentages of 30 roots colonized by AM fungi and frequencies of vesicles and arbuscules were examined and quantified using the magnified intersection method [54], and photographed using a BX61 fluorescence microscope (Olympus, Tokyo, Japan).

The seedlings’ disease resistance to hemi- and necro- trophic pathogens was characterized as follows. Seedlings infected with a *B. sorokiniana* spore suspension were uprooted at 40 dpi [55], and disease symptoms on their stems were observed. Responses of roots and stems of silenced and non-silenced wheat plants to *Ggt* were evaluated at 21 dpi [44]. For the differences in disease progression, the base sheaths of the seedlings inoculated with *B. sorokiniana* and *Ggt* harvested at 40 and 21 dpi, respectively, were fixed with 1:1 (*v*/*v*) ethanol/acetic acid for 24 h, stained with trypan blue for 6 h, then examined under a BX61 microscope.

Previous protocols of infection and microscopic examination were also applied in assessment of effects of *Bgt*-mediated powdery mildew with minor modification [56]. Leaves of wheat plants were collected 16 days after TRV-silencing (and controls), then segments were placed on 1% agar plates supplemented with 85 μM benzimidazole. The plates were incubated in a climate chamber providing constant light and 20 °C for 4 h, then the leaves were inoculated with ~200 *Bgt* conidiospores per cm^2^ of their surfaces. The *Bgt* fungus was permitted to develop on the leaf segments for 60 h, then the segments were fixed in ethanol/acetic acid (1:1, *v*/*v*), stained with Coomassie brilliant blue R250 in methanol (0.6%, *w*/*v*) for 10 s, rinsed in deionized water and microscopically examined. Samples were observed under a BX61 microscope, until at least 1000 germinated spores on leaf segments from each plant had been observed, and the spores’ germination frequencies were then calculated. Meanwhile, the phenotypes of the *Bgt* inoculated leaves were observed and photographed using a Canon EOS 600D camera (Canon Corp., Tokyo, Japan) at 7 dpi.

### 2.8. Data Analysis

The relative expression levels of genes, P concentrations of plants, and infection frequencies were calculated and significant between-treatment differences in these variables were assessed using independent samples *t*-tests and one-way analysis of variance (ANOVA) with post hoc LSD and Duncan tests (*P* < 0.01). SPSS Statistics 17.0 software (SPSS Inc., Chicago, IL, USA) was used for all the analyses, following instructions in the SPSS Survival Manual.

## 3. Results

### 3.1. Phylogenetic Relationships of PHT1 Genes in Wheat and Other Plants

**Figure 1:**
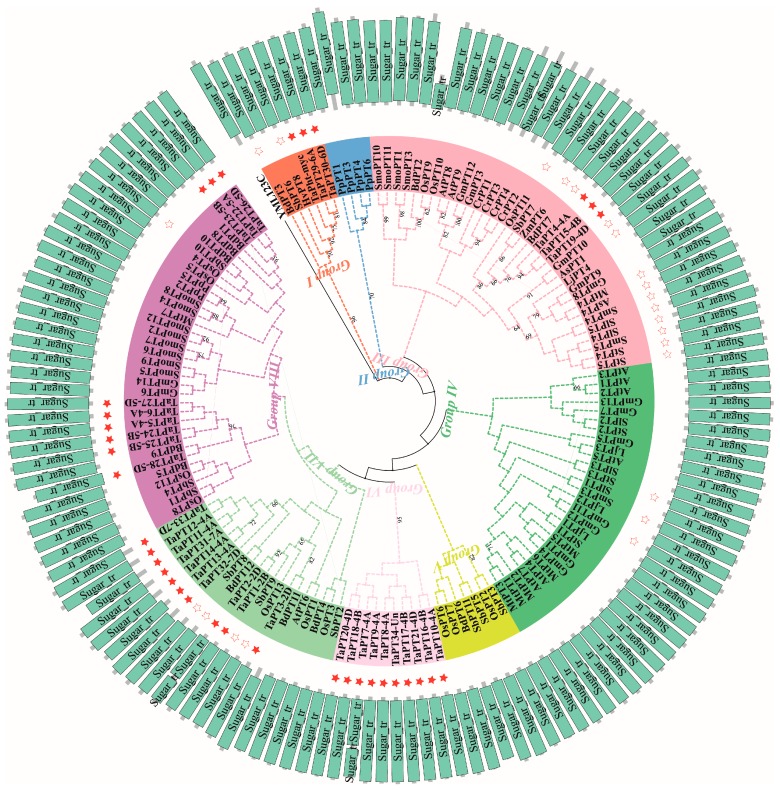
Phylogenetic tree showing conserved domains of PHT1 proteins in wheat and other plants. The phylogenetic tree was constructed by the ML method implemented in MEGA software with 1000 bootstrap replicates. The Sugar_tr domains (PF00083) were identified by Pfam and displayed in the phylogenetic tree using iTOL. PHT1 proteins in wheat and PHT1 proteins with well-known functions are highlighted by filled and empty stars, respectively. Eight subfamilies (group I, II, III, IV, V, VI, VII, and VIII) were identified and are highlighted with different colored backgrounds.

To construct a phylogenetic tree of plant PHT1 proteins, sequences of 109 PHT1 proteins from 16 plant species were collected from Phytozome or NCBI databases with the exception of rice (OsPT1-13 from RAP-DB) and *Arabidopsis* (AtPT1-9 from TAIR), representing Chlorophyte, Embryophyte, Tracheophyte and Angiosperm lineages, in addition to the 35 TaPHT1 proteins and one yeast protein (PHO84, YML123C from SDG). Then all 145 PHT1 proteins were aligned and a maximum likelihood tree was generated using MEGA7 based on full-length protein sequences with 1000 bootstrap replicates. Finally, iTOL3 was used to integrate the domain annotation for all PHT1 proteins into the phylogenetic tree. Domain analysis revealed the presence of two tandem Sugar_tr domains (PF00083) in CrPT3, TaPT2-2D and TaPT7-4A proteins, as well as variations in the PHT1 proteins’ domain lengths. Treating the YML123C protein as an outgroup, all the PHT1 proteins were grouped into eight subfamilies (groups I, II, III, IV, V, VI, VII, and VIII; Figure 1). All 35 TaPHT1 proteins were assigned to one of five of the eight subfamilies: 3, 3, 10, 10, and 9 to groups I, III, VI, VII, and VIII, respectively. Interestingly, members of groups II and VI were only found in *P. patens* and *T. aestivum*, respectively, suggesting they may be species-specific groups. Moreover, members of groups I and V were from monocotyledon plants, while members of group IV were from dicotyledons. Group VII was present in *A. thaliana* and four dicotyledonous plants, suggesting that the common ancestor of PHT1 proteins in this group arose before the division of monocotyledon and dicotyledon plants. In addition, PHT1 proteins in group III and VIII apparently originate from ancient ancestors, in the Chlorophyte and Embryophyte lineages, respectively. Importantly, there were more TaPHT1 members in five groups (I, III, VI, VII, and VIII) than of any other monocotyledon or dicotyledon plants, suggesting that these groups have specifically expanded in *T. aestivum*. Moreover, TaPht-myc, TaPT29-6A, TaPT30-6D, HvPT8, and BdPT3 clustered in group I. TaPht-myc, HvPT8 and BdPT3 are mycorrhiza- specific/inducible Pi transporters from wheat, barley and *Brachypodium distachyon*, respectively, indicating that TaPT29-6A and TaPT30-6D might also be induced by AM. TaPT4-4A, TaPT15-4B and TaPT19-4D (formerly known as TaPT12, TaPT11 and TaPT10, respectively, all of which are up-regulated in AM-colonized wheat) [31], OsPT11, ZmPT6, and BdPT7 (mycorrhiza- specific/inducible proteins from rice, maize, and *B. distachyon*, respectively) and 10 known AM-specific/inducible proteins were all found in group III, suggesting that this group might be specifically responsive to AM.

### 3.2. Sequence Features of PHT1 Genes in Wheat, Arabidopsis and Rice

**Figure 2:**
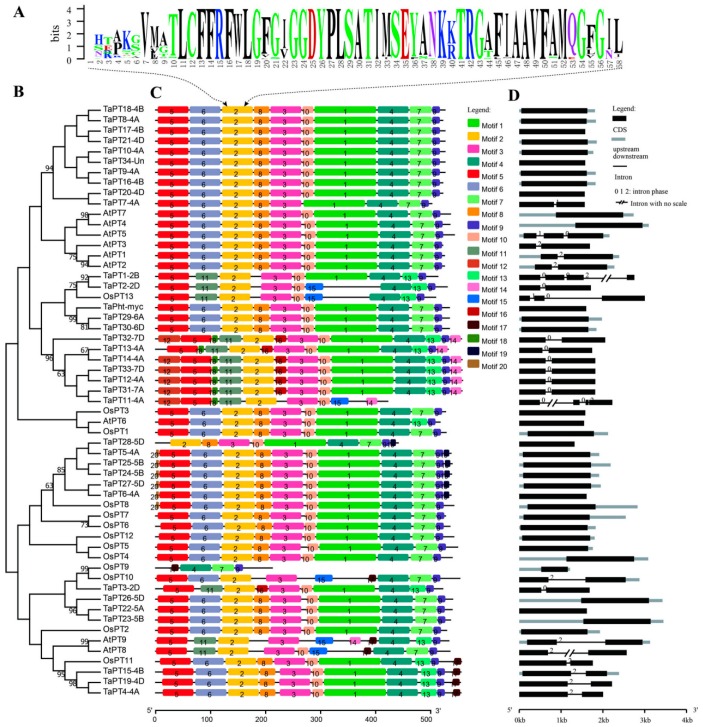
Sequence features and phylogenetic relationships of *PHT1* genes in wheat, rice and *Arabidopsis*. (**A**) The sequence logo of the conserved motif 2 was generated by Weblogo. (**B**) The phylogenetic tree was built as described in Section 2, and bootstrap values are mapped on the branches. (**C**) All the PHT1 proteins were analyzed using MEME for conserved motifs; 20 conserved motifs were named motifs 1–20. (**D**) Structures of the *PHT1* genes visualized using GSDS.

Functional and trans-membrane domains of PHT1 proteins in wheat, rice and *Arabidopsis*, 35 TaPHT1, 9 AtPHT1, and 13 OsPHT1 proteins were predicted using Pfam and TMHMM. The results show that, like previously investigated PHT1 proteins [57,58], they all have more than nine trans-membrane domains and a typical Sugar_tr domain (PF00083), suggesting that PHT1 proteins belong to the sugar transporter superfamily (Figure 1, Table S1). Interestingly, all the TaPHT1 proteins contain the core motif element (GGDYPLSATIxSE), overlapping with the conserved motif 2 (Figure 2A) visualized using Weblogo. In addition, all the TaPHT1 proteins have almost identical core motifs, except TaPT3-2D, which has alanine at position 12 rather than serine (GGDYPLSATIMAE) (Table S1).

Based on alignment of the TaPHT1, AtPHT1 and OsPHT1 proteins, a phylogenetic tree (Figure 2B) was constructed as described in Section 2. The motif architectures and gene structures of these PHT1 members were annotated within the phylogenetic context, and visualized by MEME and GSDS. Twenty putative conserved motifs from *T. aestivum*, *A. thaliana*, and *Oryza sativa* phosphate transporter protein families were identified (Figure S2, Table S2). These motifs are arranged in the PHT1 proteins in a collinear manner, and form 16 kinds of motif architecture patterns (Figure 2C). In the wheat proteins, 11 such patterns were identified, with numbers of motifs in the proteins ranging from 9 to 13, suggesting the family has diverse architecture. Each of seven kinds of motif architectures was unique to an individual TaPHT1 protein, but the others were shared by several or more TaPHT1 proteins (Figure S3). Interestingly, TaPht-myc, known as a mycorrhizal-specific protein, has a highly conserved motif architecture that was also found in 14 TaPHT1 proteins. Motif 2 was present in almost all the considered PHT1 proteins (15 TaPHT1, 13 OsPHT1 and 9 AtPHT1) but not OsPT9, although OsPT9 contains the core motif element (GGDYPLSATIxSE). According to frequencies of occurrence, all conserved motifs of wheat TaPHT1 proteins were divided into two types: common and specific. The common motifs (1–10) were identified in more than 60% of the TaPHT1 proteins, while the specific motifs (11–20) were detected in less than 35% of the proteins. In addition, motif 15 was only found in TaPT11-4A and TaPT2-2D, and motif 17 only in TaPT19-4D, TaPT4-4A and TaPT15-4B. Other specific motifs were detected in at least five TaPHT1 proteins. Importantly, five of the 10 specific motifs (12, 14, 16, 18 and 19) were only detected in wheat and are regarded here as wheat-specific motifs (Figure S3).

According to numbers and phases of introns, structures of the 35 *TaPHT1*, 13 *OsPHT1* and nine *AtPHT1* genes were classified into six types (one exon; two exons with one phase 0, 1, or 2 intron; three exons with two, phase 1 and 0, introns; and four exons with three phase 0, 0 and 2 introns; Figure 2D, Figure S4). Most of the investigated *PHT1s* (three *AtPHT1*, 10 *OsPHT1* and 21 *TaPHT1* genes) have one exon, suggesting this structure is highly conserved. In contrast, the structure of two exons with one phase 1 intron was found only in *TaPT7-4A*. Similarly, both *AtPT5* and *OsPT13* genes have three exons with two (phase 1 and 0) introns, and both *TaPT11-4A* and *TaPT1-2B* have four exons with three introns (phase 0, 0, and 2). The structural pattern of two exons with one phase 0 intron was only found in *TaHPT1* genes (eight of them). The other structural patterns are shared by three *TaPHT1*, two *OsPHT1* and five *AtPHT1* genes.

### 3.3. Chromosomal Distributions and Duplication Patterns of PHT1 Genes in Wheat

**Figure 3:**
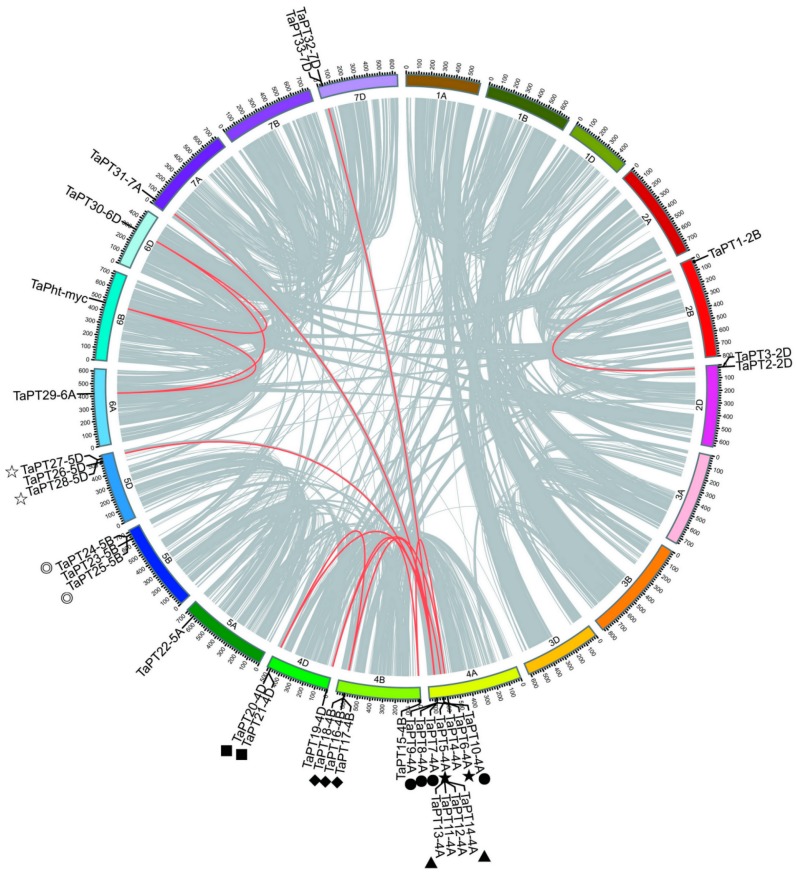
Chromosomal positions and synteny relationships of the *PHT1* genes in the wheat genome. All syntenic blocks (more than 300 anchor genes) and genes are linked by the grey lines, and segmental duplication pairs of *TaPHT1* genes are highlighted by red lines. Different symbols indicate seven tandem regions.

According to physical locations from a GFF3 file, 34 *TaPHT1* genes were visualized on the *T. aestivum* chromosomes using iTOL. These genes were unevenly distributed across all 13 chromosomes, with dramatic variation in numbers of the genes (1–11) located on each chromosome (Figure 3). Furthermore, one *TaPHT1* (*TaPT34-Un*) was present in an unassembled scaffold, and thus cannot be mapped to any particular chromosome according to the updated wheat genome used [59]. Investigation of gene duplication modes, using BLASTP and MCscanX, showed that there were 59, 168 colinear genes localized on 1, 202 syntenic blocks. Four types of duplication modes of *TaPHT1* genes were detected: singletons, dispersed, tandem and segmental. In wheat, *TaPT6-4A*, *TaPT13-4A* and *TaPT18-4B* were apparently generated by tandem duplication, and six *TaPHT1*s were amplified by dispersed duplication. Twenty *TaPHT1* genes are located in colinear regions associated with whole genome duplication (WGD) or segmental duplication events, and 19 pairs of segmental duplicates were identified among them. Seven tandem duplicate regions among 18 *TaPHT1* genes were discovered in the wheat genome, according to the following criteria: E-value 1e-10, sequence similarity > 80%, and adjacent localization on a chromosome. Interestingly, nine *TaPHT1* genes were apparently duplicated by both tandem and segmental repeats, suggesting that segmental and tandem duplication events may have been responsible for the expansion of *TaPHT1* genes in the wheat genome. To better understand evolutionary constraints of the *TaPHT1* gene family, Ka/Ks ratios of *TaPHT1* gene pairs were calculated. The Ka/Ks of all *TaPHT1* gene pairs were less than 1, indicating that the *TaPHT1* gene family may have been subject to purifying selection and functional constraints during its evolution (Table S3).

### 3.4. In silico Expression Patterns and Core Cis-Element Architecture of Wheat PHT1 Genes

Further analysis of promoters of the *TaPHT1*s showed that their 2-kb upstream regions harbor potential regulatory elements involved in Pi and AM responses (Figure S5, Table S5). Two, the P1BS (GNATATNC) and PHR1-binding cis-regulatory elements, have been previously identified in various AM-inducible Pi transporter genes [60] and play crucial roles in sorghum and flax responses to Pi starvation [61], and 27 identified *TaPHT1*s contain a P1BS element. Two consensus sequence motifs—NODCON2GM (CTCTT) and OSEROOTNODULE (AAAGAT)—have been found to be specifically active in infected cells of root nodules and arbuscule-containing cells colonized by AM [62]. Twenty-nine *TaPHT1*s harbor the NODCON2GM motif, and 17 *TaPHT1* have the OSEROOTNODULE motif. A root motif box (ATATT or AATAT) previously detected in plant PHT genes [61] was found in 34 *TaPHT1*s (all except *TaPT5-4A*). Additionally, a TTGACY W-box, WRKY transcription factor binding element, was also discovered in 30 *TaPHT1*s, which reportedly regulates expression of Pi-starvation response genes in *Arabidopsis* [63,64]. This indicates that W-boxes are involved in regulation of *TaPHT1* genes in wheat responses to P-deficiency.

### 3.5. AM Fungus Decreased the Pi Concentration and Biomass of Wheat in Vegetative Stage

Roots and shoots of wheat plants were harvested six weeks after inoculation with two species of AM fungi, *F. mosseae* and *G. versiforme*, to analyze their biomass and Pi content, and the freshly collected roots were used to determine mycorrhizal colonization. The infected plants grew slightly more slowly, in accordance with previous results [33,65,66,67,68,69]. The AM-inoculated plants also had lower biomass, and lower Pi contents of both shoots and roots, than non-inoculated controls (Table 1). Staining of fungal structures within the roots showed that the mycorrhizal symbiosis was well established in inoculated plants, with abundant fungal colonization of the root cortex and well-formed arbuscules and vesicles (Figure S6), whereas no colonization was detected in non-inoculated plants. In addition, the proportion of colonized roots was higher under low Pi than under high Pi conditions (Table 1).

### 3.6. Changes in Expression Patterns of TaPHT1 Genes in Response to AM and Fungal Pathogens

**Figure 4:**
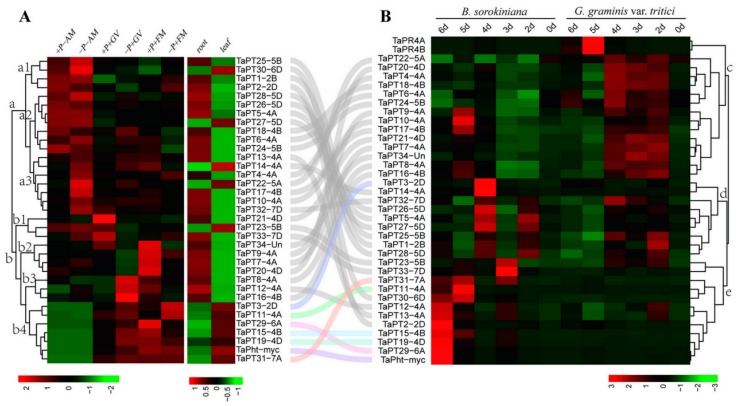
Expression profiles of 35 *TaPHT1s* under indicated treatments. (**A**) *TaPHT1s* expression patterns in roots colonized with two varieties of AM fungi after six weeks in high and low phosphorus conditions (left panel); *TaPHT1* genes’ expression patterns in roots and leaves of non-symbiotic 6-weeks-old seedlings (right panel). (**B**) *TaPHT1s* expression patterns in roots infected by *Ggt* and *B. sorokiniana* at 0–6 dpi.

First, transcript levels of *TaPHT1* genes were investigated in AM-inoculated wheat roots under high and low Pi conditions. The results indicate that the genes can be divided into clusters and subclusters designated a1, a2, a3, b1, b2, b3, and b4 (Figure 4A). Expression of genes in cluster b2 (*TaPT7-4A*, *TaPT9-4A*, *TaPT20-4D*, and *TaPT34-Un*) was only slightly increased by *F. mosseae* under high Pi conditions, and cluster b1 includes two (*TaPT21-4D*, *TaPT23-5B*, and *TaPT33-7D*) that were only induced by *G. versiforme* under high Pi conditions (Figure 4A). Cluster b3 includes three genes (*TaPT8-4A*, *TaPT12-4A*, and *TaPT16-4B*) that were slightly induced by *G. versiforme* under low Pi and *F. mosseae* under high Pi conditions. Cluster b4 contains *TaPT3-2D*, *TaPT11-4A*, *TaPT15-4B*, *TaPT19-4D*, *TaPT29-6A*, *TaPT31-7A*, and *TaPht-myc*, which were strongly expressed in roots inoculated with either of the two varieties of AM under both high and low Pi conditions, but most strongly in the presence of high Pi. All the other 18 *TaPHT1*s, in cluster a, were significantly suppressed in *F. mosseae* and *G. versiforme*-colonized roots (Figure 4A). The findings suggest that genes in clusters b and a may be mycorrhiza-specific and mycorrhiza-repressible *TaPHT1*s, respectively. We also investigated the expression of *TaPHT1* genes in non-colonized roots and leaves, and found they can be divided into two groups. Twelve, including the seven AM-inducible genes (*TaPT3-2D*, *TaPT11-4A*, *TaPT15-4B*, *TaPT19-4D*, *TaPT29-6A*, *TaPT31-7A*, and *TaPht-myc*), were mainly expressed in non-colonized wheat leaves, but the other 23 were most abundant in non-colonized wheat roots (Figure 4A).

To test the possibility that the *TaPHT1*s are transcriptionally induced by pathogenic fungi colonization, wheat roots were inoculated with the hemi-biotrophic fungus *B. sorokiniana* and necrotrophic pathogen *Ggt*. Expression of the 35 *TaPHT1*s (as well as *TaPR4A* and *TaPR4B*, which are reportedly markers of pathogen infection in wheat roots) [43,44] in roots at selected times after infection was analyzed by q RT-PCR. The genes were divided into three clusters (designated c, d and e) based on patterns of their responses to *B. sorokiniana* and *Ggt* infection. Transcription of *TaPR4A* and *TaPR4B*, was strongly increased at 5 dpi by *Ggt*, and slightly declined at 6 dpi. Cluster c and d includes 23 *TaPHT1*s that were slightly increased at 2, 3, and 4 dpi by *Ggt*. Cluster e also includes six mycorrhiza-specific/inducible genes (*TaPT11-4A*, *TaPT15-4B, TaPT19-4D*, *TaPT29-6A*, *TaPT31-7A*, and *TaPht-myc*) and the other six *TaPHT1*s showed similar expression patterns to *TaPR4A* and *TaPR4B*, significantly induced at 5 dpi, indicating that they may be involved in wheat disease resistance (Figure 4B). All the above results suggest that the 35 *TaPHT1s* were slightly or strongly induced at some time-points by interaction between wheat and *Ggt*.

Upon *B. sorokiniana* infection, the marker genes *TaPR4A* and *TaPR4B* exhibited the different expression patterns, which were significantly induced at 4 dpi and their expression slightly declined at 5 and 6 dpi (Figure 4B). Genes in cluster c (14 *TaPHT1s*) showed different expression patterns, but with transcript levels mostly increased at 4 dpi and 5 dpi. Genes in cluster d (nine *TaPHT1s*) were slightly induced at 2 dpi and their expression declined at 3 dpi, then strongly activated at 4 dpi. Cluster e also includes six mycorrhiza-specific/inducible genes (*TaPT11-4A*, *TaPT15-4B, TaPT19-4D*, *TaPT29-6A*, *TaPT31-7A*, and *TaPht-myc*) and six other *TaPHT1s* that were significantly induced at 3 dpi, 5 dpi, and 6 dpi. Thus, expression patterns of the 35 *TaPHT1s* were more varied in *B. sorokiniana*-infected roots than in *Ggt*-infected roots (Figure 4B).

**Figure 5:**
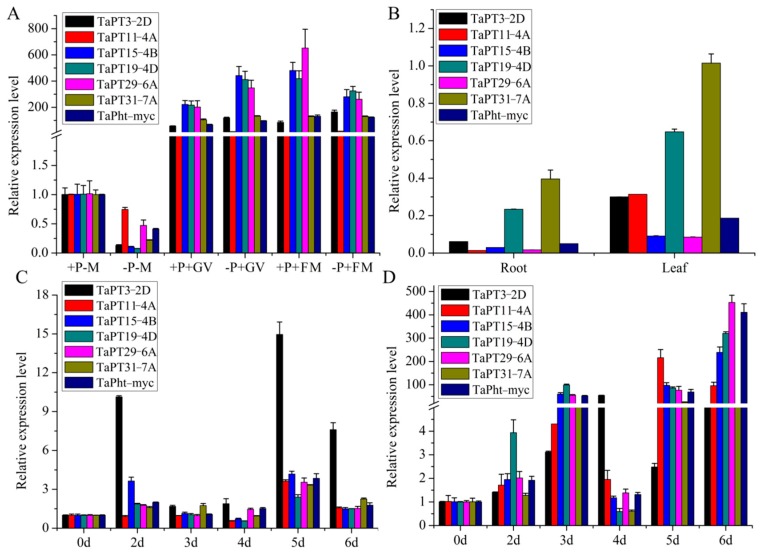
Results of real-time RT-PCR analysis of the seven mycorrhiza-inducible *TaPHT1s* under indicated treatments. (**A**) Expression levels of seven *TaPHT1s* in roots inoculated with AM fungi after six weeks in low and high phosphorus conditions. (**B**) Expression of *TaPHT1s* versus the *actin* gene in roots and leaves of non-symbiotic 6-weeks-old seedlings. (**C**) *TaPHT1* expression levels in roots infected by *Ggt* at 0–6 dpi. (**D**) *TaPHT1* expression level in roots after inoculation with *B. sorokiniana* at 0–6 dpi. Bars indicate means of three biological replications with standard errors.

Finally, expression patterns of seven *TaPHT1s* that were highly activated by both species of AM under both high and low Pi conditions (*TaPT3-2D*, *TaPT11-4A, TaPT15-4B*, *TaPT19-4D*, *TaPT29-6A*, *TaPT31-7A*, and *TaPht-myc*) were examined in more detail (Figure 5). Their expression was more strongly enhanced under low Pi than under high Pi conditions with *G. versiforme*. However, in *F. mosseae*-inoculated roots, expression levels of *TaPT15-4B*, *TaPT19-4D*, *TaPT29-6A* and *TaPht-myc* were higher under high Pi conditions. The transcription patterns are consistent with our unpublished RNA-seq data, which clearly show that *TaPT3-2D*, *TaPT11-4A*, *TaPT15-4B*, *TaPT19-4D*, *TaPT29-6A* and *TaPT31-7A* were up-regulated in wheat roots colonized by *F. mosseae*. Moreover, expression levels of *TaPT15-4B*, *TaPT19-4D* and *TaPT29-6A* were significantly higher than those of *TaPht-myc* (Figure 5A), the first identified mycorrhiza-specific *TaPHT1*. Intriguingly, in non-colonized wheat plants, all seven mycorrhiza-inducible *TaPHT1* were induced mainly in the leaves, and transcripts of *TaPT3-2D*, *TaPT11-4A*, *TaPT15-4B*, *TaPT29-6A* and *TaPht-myc* were barely detectable in the roots (Figure 5B). The findings suggest that *TaPT3-2D*, *TaPT11-4A*, *TaPT15-4B* and *TaPT29-6A* may be mycorrhiza-specific transporters, and *TaPT19-4D* and *TaPT31-7A* may be mycorrhiza-inducible transporters. In addition, transcript levels of *TaPR4A* and *TaPR4B* were respectively 946- and 1090-fold higher in *Ggt*-infected roots than in uninfected controls at 5 dpi (Figure S7A), confirming interaction between the wheat plants and fungus. q RT-PCR analysis detected no obvious differences in relative expression levels of any of the seven AM-inducible *TaPHT1* between *Ggt*-infected and uninfected roots, except for *TaPT3-2D*, transcript levels of which were enhanced 15-fold at 5 dpi by *Ggt* infection (Figure 5C).

Interestingly, *B. sorokiniana* infection induced substantial changes in expression levels of all seven AM-specific/inducible *TaPHT1*s at 3, 5, 6 dpi. Most notably, a 453-fold increase in expression of *TaPT29-6A* at 6 dpi (Figure 5D) as well as 48- and 59-fold increases in expression levels of *TaPR4A* and *TaPR4B*, respectively, were observed at 3 dpi (Figure S7B). Based on the above results, *TaPT29-6A* was selected as representative AM-specific *TaPHT1* for functional verification.

### 3.7. TaPT29-6A Is Localized in Plasma Membrane

**Figure 6:**
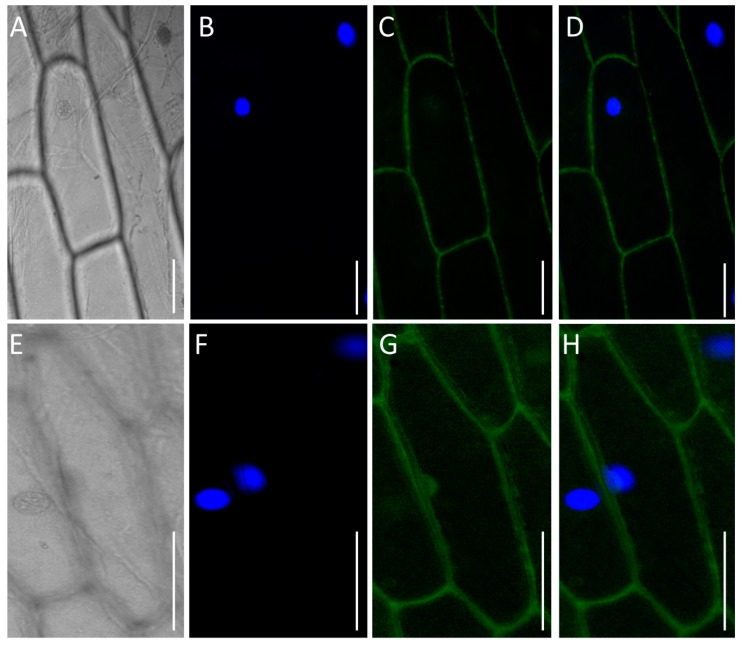
Subcellular localization of TaPT29-6A/GFP protein. Images showing onion epidermal cells expressing TaPT29-6A/GFP (**A**–**D**) and empty vector (**E**–**H**). Bright field illumination (**A**,**E**); DAPI-stained nuclei (**B**,**F**); UV excited fluorescence images (**C**,**G**); merged images of DAPI and fluorescence (**D**,**H**). Scale bar = 25 µm.

TMHMM Server V. 2.0, used to predict transmembrane helices in proteins, indicated that TaPT29-6A has 12 transmembrane domains (Table S1). To verify TaPT29-6A′s subcellular localization, a sequence encoding the full-length protein was fused to the 5′ terminus of the green fluorescent protein (*GFP*) gene in the pSAT6-*GFP* vector, with expression driven by the constitutive *CaMV* 35S promoter. The TaPT29-6A protein was transiently transferred into onion epidermal cells through *Agrobacterium tumefaciens*-mediated transformation. Microscopic imaging of fluorescent signals from the TaPT29-6A-GFP fusion protein showed that TaPT29-6A was localized in the plasma-membrane, while fluorescence signals from the control pSAT6-GFP vector were ubiquitous in the cells (Figure 6). These results confirm that TaPT29-6A is a membrane-localized protein, like GmPT1/2, MtPT3/5 and AsPT1/4 [23,70,71].

### 3.8. TaPT29-6A Is Required for Symbiotic and Non-Symbiotic Pi Uptake

**Figure 7:**
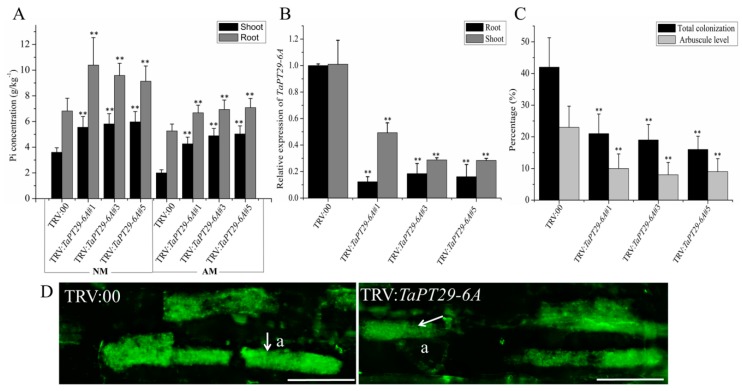
Arbuscular mycorrhizal phenotypes of *TaPT29-6A*-silenced plants. Fluorescence microscopic images showing arbuscules in wheat roots silenced with a *TaPT29-6A* partial sequence and controls infected with the TRV:00 vector. (**A**) Pi concentrations in indicated tissues of *TaPT29-6A*-silenced and control plants in the presence and absence of *F. mosseae* at 42 dpi. (**B**) Relative transcript levels of *TaPT29-6A* in control and silenced plants. Bars indicate means of three biological replicates with standard errors. (**C**) Levels of *F. mosseae* colonization and arbuscules at 42 dpi in *TaPT29-6A*-silenced and control plants. (**D**) Wheat roots of *TaPT29-6A*-silenced and control plants stained with WGA-Alexafluor 488 to view *F. mosseae* arbuscules in their cells at 6 wpi. Double asterisks indicate significant differences relative to TRV:00 plants (*P* ≤ 0.01 according to *t* tests). a = arbuscules. Bars = 12.5 µm. NM, non-colonized; AM, colonized.

To characterize roles of *TaPT29-6A* in phosphate transport *in planta*, shoot and root tissues of *TaPT29-6A* knockdown lines were analyzed to determine their Pi levels in the absence and presence of *F. mosseae*. Interestingly, uninfected plants expressing a VIGS construct targeting *TaPT29-6A* had much higher Pi concentrations in both shoots and roots than unsilenced controls (Figure 7A). Furthermore, *TaPT29-6A* silencing elevated Pi concentrations in both roots and shoots of AM-inoculated roots and shoots (Figure 7A). In addition, the Pi concentration was higher in roots than in shoots of both *TaPT29-6A*-silenced and control plants. Moreover, in the VIGS and control plants, the concentration of Pi in roots was much higher than that of Pi in shoots. All these results indicate that *TaPT29-6A* might serve as a negative regulator for Pi uptake in both symbiotic and non-symbiotic pathways.

### 3.9. TaPT29-6A Silencing Decreased Wheat Colonization by AM Fungus and Increased Susceptibility to Pathogenetic Microbes

**Figure 8:**
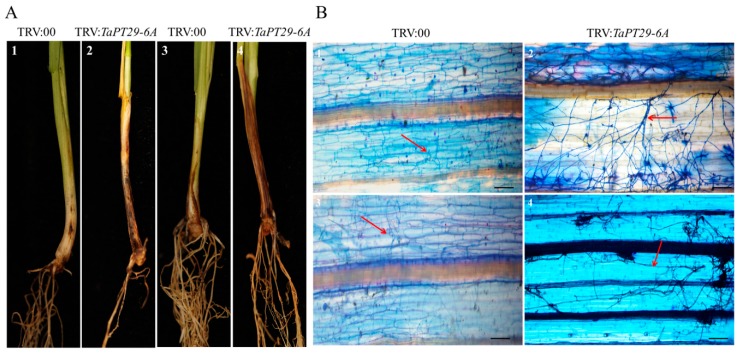
Silencing *TaPT29-6A* increased susceptibility to *Ggt* and *B. sorokiniana*. (**A**) Typical infection phenotypes of 16 days after silencing control plants and *TaPT29-6A*-silenced plants after inoculation with *Ggt* (1–2) and *B. sorokiniana* (3–4) for 21 days and 40 days. (**B**) Microscopic images of *Ggt* (1–2) and *B. sorokiniana* (3–4) hyphae on the base leaf sheaths of *TaPT29-6A* VIGS and TRV:00 plants. Red arrows indicate the hyphae of *Ggt* and *B. sorokiniana* pathogens. Scale bar = 25 µm.

As *TaPT29-6A* was highly induced by AM and detrimental fungal pathogens (Figure 5A,C,D), TRV-VIGS silencing of the mycorrhiza-specific gene *TaPHT1* was also used to evaluate its potential roles in wheat responses to various microbes. Knockdown efficiency of *TaPT29-6A* was confirmed by q RT-PCR analyses showing that its transcript levels were much lower in the silenced roots and shoots than in controls (Figure 7B). Following a published whole-plant silencing method [47], *TaPT29-6A*-silenced (and control) seedlings were generated and inoculated with *F. mosseae* after 6 weeks. *TaPT29-6A*-silenced roots had lower levels of AM and arbuscules colonization than controls (Figure 7C). However, they also had mature arbuscules (Figure 7D), and no differences in arbuscule morphology were detected between control and VIGS-silenced plants (Figure 7D). In addition, roots of *TaPT29-6A*-silenced seedlings were inoculated with the two root pathogens 16 days after silencing. The *TaPT29-6A*-VIGS lines exhibited significantly enhanced susceptibility to the two root pathogens *Ggt* and *B. sorokiniana*, at 21 and 40 dpi, respectively (Figure 8A). Moreover, microscopic observation revealed more *Ggt* and *B. sorokiniana* hyphae at the base sheaths of *TaPT29-6A*-VIGS lines than on TRV:00 plants (Figure 8B).

**Figure 9:**
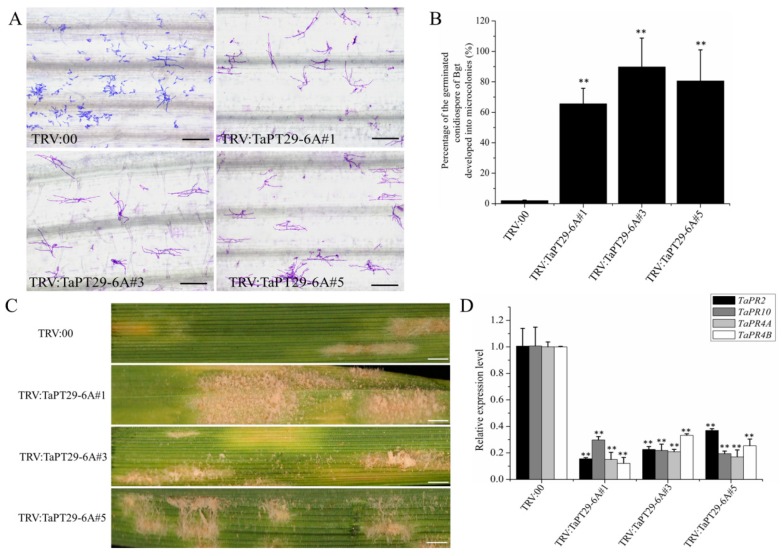
Silencing *TaPT29-6A* enhanced susceptible to *Bgt*. (**A**) Microscopic images showing *Bgt* microcolony formation on leaves of TRV:00 plants and *TaPT29-6A*-silenced plants, 60 h after inoculation. Scale bar = 12.5 µm. (**B**) Percentages of germinated *Bgt* conidiospores on the TRV:00 plants and *TaPT29-6A*-silenced plants 60 h after infection. (**C**) Macroscopic phenotypes of *Bgt* infection on leaves of TRV:00 plants and *TaPT29-6A*-silenced plants. Scale bar = 5 mm. (**D**) Results of qRT-PCR analysis of relative transcript levels of *TaPR4A/B* and *TaPR2/10* in *TaPT29-6A*-silenced and TRV: 00 plants. Double asterisks indicate significant differences between the plants at *P* ≤0.01, according to a *t* test.

We subsequently assessed *TaPT29-6A*-silenced lines’ resistance to *Bgt*, which has a completely different life cycle from *Ggt* and *B. sorokiniana*, but is considered a biotrophic pathogen, like AM fungi. At 16 days after virus inoculation, fourth leaves of the plants (and controls) were detached and infected with fresh spores of *Bgt*. The leaves were examined under a microscopic 60 h after infection with *Bgt*, and percentages of germinated conidiospores that developed into microcolonies were calculated. There were significantly more *Bgt* microcolonies on leaves from *TaPT29-6A*-silenced lines than on leaves inoculated with the TRV:00 vector (Figure 9A,B). Clear mycelial colonies were macroscopically observed on leaves of *TaPT29-6A*-silenced plants, while few were found on leaves of control plants (Figure 9C), in accordance with the microscopy results. These results indicate that *TaPT29-6A* silencing weakens resistance to powdery mildew caused by *Bgt*. To examine whether silencing *TaPHT1* could affect the expression levels of defense response genes, *TaPR4A/B* [45,46] and *TaPR2/10* [72,73] were chosen as marker genes for defense responses to necrotrophic and biotrophic pathogens. In addition, qRT-PCR analysis showed that transcript levels of the *TaPR4A/B* and *TaPR2/10* marker genes for defense responses to necrotrophic and biotrophic pathogens were significantly lower in the *TaPT29-6A*-silenced plants than in the controls (Figure 9D), in accordance with the susceptible phenotypes of VIGS plants. In conclusion, all the above results indicate that *TaPT29-6A* may be a positive regulator in wheat immunity signaling pathways.

## 4. Discussion

Little is known about roles of wheat PHT1 Pi transporters in AM symbiosis-mediated Pi uptake. Previous studies have identified multiple AM-specific/inducible PHT1 transporters in *O. sativa* [17,26], *Hordeum vulgare* [25], *Zea mays* [25], *Sorghum bicolor* [61], *B. distachyon* [74], *T. aestivum* [25,33], and both leguminous [18,23,75] and solanaceous plant species [24,27,29]. However, there have been few functional analyses of wheat AM-inducible PHT1 transporters, although *TaPHT1* gene expression sites, regulation by mineral nutrition, and responses to P availability have been recently examined in genome-wide analyses [76,77]. In this study, we comprehensively surveyed *TaPHT1* genes, using a high-quality, fully annotated and ordered bread wheat genome sequence [59], and examined their roles under both low and high Pi conditions in the presence and absence of various types of pathogenic fungi. We also verified the function of one identified gene, by TRV-VIGS, in wheat responses to the tested organisms, showing that it is not only involved in P uptake through the AM symbiosis pathway but also in interactions between wheat and various pathogenic fungi. 

### 4.1. Negative Effects of AM Fungi on Wheat

AM plants have evolved two P absorption pathways: the direct and mycorrhizal pathways [20]. Many plants that form symbioses with AM show clear positive responses to colonization, including cereal crops [78,79] and various other plants including *Medicago truncatula* [22], *Linum usitatissimum* [80], and *Astragalus sinicus* [23]. However, some plant species show negative responses to AM colonization, including growth depression in wheat and barley [33,65,66,81,82]. In accordance with previous studies, in an early growth stage (~6 weeks) the biomass and P contents of wheat plants we studied were lower in AM-colonized plants than in non-colonized controls. However, this does not necessarily mean that the AM made no contribution to P uptake, because (for example) ^32^P has been detected in all AM-colonized wheat plants, but not in non-colonized controls, in studies of tracer-based Pi uptake [68]. The early AM-induced wheat growth depression, which often disappears at maturity, is probably due to C demands of AM reducing the C available for host plants’ growth [81], and/or levels of soluble sugars in their leaves [67,83]. Moreover, there are variations in plants’ responses associated with differences in plant taxa, AM taxa and growth stages [81,84,85]. Previous research has also indicated that negative plant responses to AM colonization are not related to the percentage of AM colonization, but suppression of P uptake by the direct pathway, without full compensation by the mycorrhizal pathway, may contribute to growth depression [20]. The negative growth response can be mitigated by increasing the density of host plants and decreasing the AM biomass [81]. There have also been reports of positive effects of AM colonization on growth and P uptake at maturity [86,87], and under appropriate circumstances it can have positive effects on agronomic traits in wheat [88,89,90]. Furthermore, it may be possible to engineer crop responses to increase the activity of the direct pathway, thereby promoting their growth. 

### 4.2. PHT1 Transporters in Wheat

We identified 35 *TaPHT1* genes in an updated wheat genome, and analyzed them in detail in terms of phylogenetic relationships, sequence features, duplication modes and selection pressure. All the corresponding proteins have a highly conserved Sugar_tr domain (PF00083), and the core sequence “GGDYPLSATIxSE” located in motif 2. The phylogenetic analysis showed that the 35 *TaPHT1*s can be classified into five groups, and *PHT1* genes in group III are closely related to those of groups I and II (Figure 1), forming an ancient and polyphyletic clade originating from a Viridiplantae ancestor. Moreover, the ancestor of *PHT1* genes of group VIII was apparently of Embryophyte lineage, while groups IV, V, VI, and VII seem to be dicotyledon-, monocotyledon-, wheat- and angiosperm-specific, respectively. These findings suggest a complex evolutionary history of *TaPHT1*s, which is supported by many previous studies [1,23,30,91]. Interestingly, some *PHT1* genes with similar biological functions are distributed across different groups [22,23,25,26,29,61,74,75]. In group I, AM-specific/inducible genes including *TaPht-myc*, *BdPT3* and *HvPT8* were found, and wheat homologs (*TaPT29-6A* and *TaPT30-6D*) predicted to be induced by AM (Figure 1) [25,74]. We subsequently found that *TaPht-myc* and *TaPT29-6A* were highly expressed in *G. versiforme* and *F. mosseae*-colonized roots, but *TaPT30-6D* transcription was repressed by the two AM fungi (Figure 5A), implying that *TaPT30-6D* may be induced by other species of AM and/or functions of these three genes have diversified in wheat. Similarly, 13 *PHT1* genes with well-known functions were present in group III, and homologs in wheat, including *TaPT4-4A*, *TaPT15-4B* and *TaPT19-4D* (formerly known as *TaPT12*, *TaPT11*, and *TaPT10*), might have similar functions. In addition, these three *TaPHT1* genes were generated by duplication events, and evidence of phosphate transporter genes’ duplication has also been found in eggplant (*SmPT4* and *SmPT5*), tomato (*SlPT4* and *SlPT5*) and potato (*StPT4* and *StPT5*) [29]. In summary, these conserved proteins of group III share an ancient common ancestor and participate in mycorrhizal symbiosis in monocotyledons and dicotyledons [26,29]. Moreover, four known AM-inducible *PHT1* transporters (*BdPT12*, *SbPT8*/*9*, and *OsPT13*) [26,29,61] and 10 *TaPHT1s* clustered in group VII, three of which (*TaPT3-2D*, *TaPT11-4A* and *TaPT31-7A*) were both highly induced by *G. versiforme* and *F. mosseae* in high and low Pi conditions (Figure 5A). Speculatively, the other seven *TaPHT1* genes may respond to other species of AM, like *SbPT8* and *SbPT9*, which are reportedly only induced by particular kinds of AM [61]. 

In further phylogenetic analysis, *TaPHT1* sequence features were examined in detail at nucleotide and protein levels. Clearly, *PHT1* genes in the same group have similar motif architectures and exon-intron structures, as also found in *Arabidopsis* [12], rice [6], maize [92] and poplar [30], suggesting that sequence patterns are highly conserved among different plant species (Figure 2). Motif analysis showed that PHT1 proteins in wheat (11 types) have many more types of motif architectures than those in rice (seven types) and *Arabidopsis* (four types), and diverse motif architectures may be associated with rapid evolutionary divergence in *TaPHT1* genes after wheat genome duplication events [59,93]. Moreover, additional motifs (including motifs 12, 14, 16, 18 and 19) were observed in TaPHT1 proteins, which are specific to wheat and may be responsible for the diversity of motif architectures (Figure S3). With respect to exon number and intron phase, two kinds of gene structures are highly conserved in wheat, rice and *Arabidopsis*. Three new gene structures were found only in wheat, and one was only found in rice and *Arabidopsis* (Figure S3). In summary, there were more motif architectures than gene structures in the wheat PHT1 family, which could be related to functional divergence of TaPHT1 proteins.

Physical mapping showed that *TaPHT1* genes were unevenly distributed on 13 of the 21 wheat chromosomes. Synteny analysis revealed that their expansion may have involved segmental and tandem duplication, and evidence of these amplification modes has also been found in maize and soybean [92,94]. In addition, Ka/Ks values of 14 tandemly and 19 segmentally duplicated gene pairs strongly suggest that each duplicated gene pair has been subject to purifying selection, implying these gene pairs are under functional constraints. According to a published timeline of wheat evolution [95], divergence times of the 33 gene pairs ranged from 0.96 to 21.16 Mya, before divergence of the A, B, D genome from a common progenitor. In addition, putative divergence times of five gene pairs are less than 0.5 Mya, suggesting they separated before emergence of wild allotetraploid wheat (Table S3).

### 4.3. Transcriptional Analysis of TaPHT1 Gene Expression

*TaPht-myc*, the first mycorrhizal-specific *TaPHT1* to be identified, was highly expressed in wheat roots colonized by *G. versiforme* and *F. mosseae* in both high and low Pi conditions, as previously reported [25]. However, there were major differences in expression patterns of the 35 *TaPHT1*s in responses to symbiotic and pathogenic colonizers. Four novel mycorrhizal-specific/inducible genes (*TaPT3-2D*, *TaPT11-4A*, *TaPT29-6A*, and *TaPT31-7A*) were identified in this study, plus *TaPT15-4B* and *TaPT19-4D* (formerly known as *TaPT11* and *TaPT10*, respectively). All of these genes were strongly expressed in wheat roots inoculated with *G. versiforme* and *F. mosseae* under high and/or low Pi conditions. However, while *TaPT4-4A* (formerly known as *TaPT12*), *TaPT15-4B* and *TaPT19-4D* are reportedly induced in wheat roots colonized by *G. intraradices* [33], we found that *TaPT15-4B* and *TaPT19-4D* were induced in AM-inoculated wheat roots but *TaPT4-4A* was suppressed, suggesting that *TaPT4-4A* might have race-specificity. Some of the other genes were induced in wheat roots exposed to low Pi, and might be involved in the Pi direct pathway, while others were down-regulated in AM-inoculated roots. This is consistent with previous predictions from molecular and physiological observations that AM colonization leads to significant changes in plants’ Pi uptake, including not only up-regulation of AM-inducible phosphate transporters, but also down-regulation of P transporter genes involved in the direct Pi pathway [25].

To investigate responses of *TaPHT1s* expression to colonization by microbes in more detail, we challenged roots of wheat plants with several pathogenic fungi that use different strategies for invading and obtaining host nutrients. In similar studies with rice plants, the AM-specific gene *OsPT11* was not induced by *Fusarium moniliforme* or *Rhizoctonia solani*, indicating that *OsPT11* is specific to mycorrhizal colonization [17]. Interestingly, inoculation of wheat roots by the necrotrophic pathogen *Ggt* induced increases in expression of seven mycorrhizal-specific/inducible *TaPHT1*s at 5 dpi, especially *TaPT3-2D* (a 15-fold increase), as shown in Figure 5C. In wheat roots, infection by the hemi-biotrophic fungus *B. sorokiniana* increased expression levels of seven mycorrhizal-specific/inducible *TaPHT1* more highly than *Ggt*, particularly *TaPT29-6A* (Figure 5D). Our subsequent functional verification corroborated the involvement of *TaPHT1*s not only involved in AM symbiosis, but also interactions between wheat and pathogenic fungi, demonstrating that *TaPHT1* might have dual functions in symbiosis and immunity signaling.

### 4.4. TaPT29-6A Is Required for Pi Uptake in Direct and Symbiotic Pathways, and Knock-Down of TaPT29-6A Reduces Colonization of AM Fungus

As representative AM-specific/inducible *PHT1* genes in monocots, functions of *OsPT11* and *OsPT13* have been systematically analyzed in rice. In wild-type plants without inoculation of AM fungus, P concentrations in shoot and root tissues were similar to those in *OsPT11* and *OsPT13* mutant lines, indicating that *OsPT11* and *OsPT13* have no effect on Pi nutrition in the absence of symbiosis. However, AM colonization induced reductions in P concentrations in *OsPT11* mutant plants, relative to those in both *OsPT13* mutant lines and control plants, demonstrating that *OsPT11* but not *OsPT13* is essential for symbiotic Pi uptake [26]. Similar differences in expression patterns have been observed in orthologue genes of *OsPT11* in leguminous (*MtPT4*; *AsPT4*) [22,23] and solanaceous plants (*SlPT4/5*; *StPT4/5*) [29]. In marked contrast, we found that *TaPT29-6A*-silenced wheat lines had higher Pi concentrations than the TRV: 00 controls without AM inoculation. This suggests that *TaPT29-6A* might be involved in direct Pi uptake, unlike known PHTs, such as *OsPT11*/*13*, *AsPT1/4* and other homologous genes, which have been found to have no effect on Pi nutrition in the absence of symbiosis [23,26]. In addition, AM colonization increased Pi concentrations in *TaPT29-6A*-silenced lines, but reduced them in plants with RNAi-silenced *OsPT11* or *AsPT1*, and had no effects on Pi levels in *OsPT13* mutants, relative to wild-type controls [23,26]. These findings suggest that *TaPT29-6A* might be a phosphate transporter that acts in a novel manner, suppressing Pi uptake via direct and symbiotic pathways, and the mechanism whereby *TaPT29-6A* regulates Pi uptake in the presence and absence of AM symbiosis warrants further attention.

Furthermore, levels of both colonization by AM and arbuscules were lower in *TaPT29-6A*-silenced plants than in controls, but mature arbuscules were observed in them. This is consistent with previous observations that *OsPT11* and *OsPT13* mutants had smaller and less intensely branched arbuscules than controls [26]. However, RNA interference with *MtPT4* [22] and *AsPT1/4* [23], reportedly led to degeneration and premature death of arbuscules, terminating development of symbiosis. Interestingly, dicots, especially solanaceous species, have at least two AM-inducible *PHT1* genes (*SlPT4* and *SlPT5*), and there is a high degree of functional redundancy between them [29,96]. It should be noted that the segment used to construct the TRV:*TaPT29-6A* vector was identical to sequences in *TaPT29-6A*, *TaPht-myc*, and *TaPT30-6D*. Whether these three Pi transporter genes and the other AM-specific/inducible *TaPHT1* (*TaPT3-2D*, *TaPT11-4A*, *TaPT15-4B*, *TaPT19-4D*, and *TaPT31-7A*) have similar genetic redundancy requires verification through silencing with characteristic sequences of target genes.

### 4.5. TaPT29-6A Has Dual Functions in AM Symbiosis and Immunity Signaling

Chitin and its derivatives, chitooligosaccharides (COs), the main components of fungal cell walls, are microbial associated molecular patterns (MAMPs) that are perceived by pattern recognition receptors (PRRs) of plants [97]. COs (short- and long-chain) and lipochitooligosaccharides (LCOs) are respectively produced by pathogenic fungi and symbiotic AM [98,99]. The high similarities between pathogenic chitin signals and symbiotic LCOs signals raise questions about how plants discriminate between CO-containing molecules that induce immunity from those that trigger symbiosis, eventually leading to plant decisions to let friends (symbionts) enter or to shut out foes (pathogens) [100]. Various LysM-domain-containing receptor-like protein kinases secreted by plants activate immune responses or symbiosis signaling pathways [101]. Recent finding have shown that CERK1 proteins, isolated from rice [102] and *M. truncatula* [103,104] have dual functions as co-receptors in immunity and symbiosis. These findings indicate that pathogens might hijack components of symbiotic machinery, which may help plants to adjust quickly to complex and changing environments [105], like AM-specific phosphate transporter *TaPT29-6A*.

Previous studies of phosphate transporters have mainly focused on their roles in absorbing and reallocating inorganic phosphate in plants, and roles of phosphate transporters in plant defense have rarely been reported, except for *PHT4* genes in *Arabidopsis* [106]. Knocking out Golgi-located phosphate transporter *pt4;6* in *Arabidopsis* reportedly increases resistance to the bacterial pathogen *Pseudomonas syringae* strain DC3000 [107], and *AtPT4;1* also appears to be a negative regulator of plant defenses against *Pseudomonas* strains [106]. Our work revealed dual functions of *TaPT29-6A* in mycorrhizal and immunity signaling, and that its silencing led to a decrease in mycorrhizal colonization, but increases in susceptibility to various microbes, including biotrophic, hemi-biotrophic and necrotrophic pathogens, like *OsCERK1* [102]. This is the first report that PHT1 phosphate transporters are involved in wheat’s disease resistance mechanisms, indicating that they also play roles in responses to biotic stress. Expression patterns of diverse plant defense marker genes (*PRs*) are reportedly altered in *pt4* mutant plants [106], and *AtPT4; 1* acted as an upstream gene in salicylic acid signaling [106]. Similarly, transcript levels of *PR* genes significantly decreased in the *TaPT29-6A*-silenced plants, suggesting that *TaPT29-6A* might be involved in plant defenses via plant hormone pathways. This possibility requires attention in future research.

## 5. Conclusions

Taken together, the data provide detailed information about the chromosomal organization, phylogenetic evolution and expression patterns of *PHTs* in wheat. In addition, the functional characterization of *TaPT29-6A* offers new insights into roles of phosphate transporters in wheat symbioses and immunity. Future studies should include analyses of effects of over-expressing and silencing the other AM-specific/inducible phosphate transporter genes in wheat, and exploration of their potential functions in wheat’s phosphate transport and responses to microbes. This will help to strengthen our understanding of the multiple roles of AM-specific/inducible PHT1s in symbiotic phosphate uptake in wheat, of the establishment of AM symbiosis and of immune signaling pathways.

## Figures and Tables

**Table 1:** AM colonization, growth and phosphorus (P) response of wheat inoculated with *F. mosseae*, *G. versiforme* or non-mycorrhizal for 6 weeks.

Sample	P Treatment (µM)	AM Treatment	Colonization (%)			Total Biomass ^1^ (g Per Plant)	P Concentration ^1^ (g kg^−1^)	
			Total	Arbuscule	Vesicle		Shoot	Root
+P − AM	500	-	-	-	-	1.2 ± 0.11^a^	6.7 ± 0.8 ^a^	7.5 ± 0.9 ^a^
+P + GV	500	*G. versiforme*	38 ± 2.9	17 ± 1.6	13 ± 1.0	1.1 ± 0.07 ^b^	5.5 ± 0.4 ^b^	6.4 ± 1.1 ^b^
+P + FM	500	*F. mosseae*	41 ± 3.2	21 ± 1.9	11 ± 1.1	1.0 ± 0.08 ^c^	5.6 ± 0.7 ^c^	6.6 ± 0.8 ^c^
−P − AM	5	-	-	-	-	0.9 ± 0.21 ^d^	5.6 ± 0.5 ^d^	5.9 ± 1.0 ^d^
−P + GV	5	*G. versiforme*	45 ± 3.1	22 ± 1.8	15 ± 0.9	0.8 ± 0.15 ^e^	5.1 ± 0.5 ^e^	5.1 ± 0.8 ^e^
−P + FM	5	*F. mosseae*	49 ± 4.3	28 ± 2.3	12 ± 0.7	0.8 ± 0.45 ^e^	5.0 ± 0.6 ^f^	4.7 ± 0.9 ^f^

^1^ Values are means ± SEM of three replicates. Values with the lowercase letters (a, b, c, d, e, f) in each column are significantly different (*P* < 0.05), and the same lowercase letter (e) indicates no significant differences.

## Supplementary Materials

The following are available online at https://www.mdpi.com/2073-4409/8/5/490/s1, Tables S1–S5; Figures S1–S7.
