# Supplementary material for: Integrative Analysis of the Wheat PHT1 Gene Family Reveals A Novel Member Involved in Arbuscular Mycorrhizal Phosphate Transport and Immunity
Source: Cells. 2019 May 22;8(5):490. doi: 10.3390/cells8050490 (PMC6562588; doi:10.3390/cells8050490)
Supplement: Supplementary file 1 [file cells-08-00490-s001.pdf]

Article

Integrative analysis of the wheat *PHT1* gene family reveals a novel member involved in arbuscular mycorrhizal phosphate transport and immunity

Yi Zhang[1,2,3], Lizong Hu[2,3], Deshui Yu[2,3], Kedong Xu[2,3], Ju Zhang[2,3], Xiaoli Li[2,3], Pengfei Wang[1], Guo Chen[2,3], Zhihui Liu[2,3], Chunfeng Peng[2,3], Chengwei Li[1,2,3,4*], and Tiancai Guo[1*]

[1]The Collaborative Innovation Center of Henan Food Crops, Henan Agricultural University, Zhengzhou, 450002, China

[2]Key Laboratory of Plant Genetics and Molecular Breeding, Zhoukou Normal University, Zhoukou, 466001, China

[3]Henan Key Laboratory of Crop Molecular Breeding & Bioreactor, Zhoukou, 466001, China

[4]Henan Engineering Research Center of Grain Crop Genome Editing, Henan Institute of Science and Technology, Xinxiang, 453003, China

* Correspondence: E-mail: lichengweihist@163.com, guotiancai@henau.edu.cn

Table S1. Characteristics of the wheat PHT1 gene family.

Gene name	Gene stable ID	Chromosome name	Gene start (bp)	Gene end (bp)	Strand	Length of ORF sequence (bp)	Length of amino acids sequence (AA)	Transmembran domains	Best blast hit with *Oryza sativa* PHT1(Identity in %)	Best blast hit with *O. sativa* PHT1(E-value)	PHT1 signature GGDYPLSATIxSE
TaPT1-2B	TraesCS2B02G059100	2B	28725167	28729269	-1	1530	509	11	OsPT13(71)	0	GGDYPLSATIMSE
TaPT2-2D	TraesCS2D02G045600	2D	16588730	16590442	-1	1590	529	11	OsPT13(70)	0	GGDYPLSATIMSE
TaPT3-2D	TraesCS2D02G045700	2D	16658676	16660359	1	1566	521	12	OsPT4(60)	0	GGDYPLSATIMAE
TaPT4-4A	TraesCS4A02G301100	4A	598435208	598437212	-1	1584	527	12	OsPT8(60)	0	GGDYPLSATIMSE
TaPT5-4A	TraesCS4A02G359900	4A	632989596	632991508	1	1515	504	11	OsPT8(87)	0	GGDYPLSATIMSE
TaPT6-4A	TraesCS4A02G360000	4A	632998265	632999875	-1	1611	536	11	OsPT8(87)	0	GGDYPLSATIMSE
TaPT7-4A	TraesCS4A02G416300	4A	685939441	685941006	-1	1506	501	11	OsPT2(77)	0	GGDYPLSATIMSE
TaPT8-4A	TraesCS4A02G416400	4A	685996965	685998797	-1	1566	521	12	OsPT2(81)	0	GGDYPLSATIMSE
TaPT9-4A	TraesCS4A02G416500	4A	686138336	686140164	-1	1578	525	12	OsPT2(81)	0	GGDYPLSATIMSE
TaPT10-4A	TraesCS4A02G416600	4A	686168444	686170214	-1	1578	525	12	OsPT2(81)	0	GGDYPLSATIMSE
TaPT11-4A	TraesCS4A02G417900	4A	687813492	687832443	1	1671	556	12	OsPT13(65)	5.49E-179	GGDYPLSATIMSE
TaPT12-4A	TraesCS4A02G418000	4A	687892533	687894351	1	1674	557	10	OsPT13(64)	0	GGDYPLSATIMSE
TaPT13-4A	TraesCS4A02G418300	4A	688325963	688327706	-1	1596	531	9	OsPT13(63)	0	GGDYPLSATIMSE
TaPT14-4A	TraesCS4A02G418400	4A	688373566	688375384	-1	1671	556	10	OsPT13(62)	0	GGDYPLSATIMSE
TaPT15-4B	TraesCS4B02G013200	4B	9385719	9388103	1	1665	554	12	OsPT8(60)	0	GGDYPLSATIMSE
TaPT16-4B	TraesCS4B02G317000	4B	606816672	606818496	-1	1566	521	12	OsPT2(81)	0	GGDYPLSATIMSE

TaPT17-4B	TraesCS4B02G317100	4B	606866119	606867696	-1	1578	525	12	OsPT2(81)	0	GGDYPLSATIMSE
TaPT18-4B	TraesCS4B02G317200	4B	606907389	606909200	-1	1578	525	11	OsPT2(81)	0	GGDYPLSATIMSE
TaPT19-4D	TraesCS4D02G010900	4D	5288175	5290392	-1	1584	527	12	OsPT8(60)	0	GGDYPLSATIMSE
TaPT20-4D	TraesCS4D02G313700	4D	479635733	479637298	-1	1566	521	12	OsPT2(81)	0	GGDYPLSATIMSE
TaPT21-4D	TraesCS4D02G313800	4D	479674652	479676505	-1	1578	525	12	OsPT2(81)	0	GGDYPLSATIMSE
TaPT22-5A	TraesCS5A02G460300	5A	640724647	640726263	1	1617	538	12	OsPT8(81)	0	GGDYPLSATIMSE
TaPT23-5B	TraesCS5B02G470100	5B	643459208	643462655	1	1608	535	12	OsPT8(80)	0	GGDYPLSATIMSE
TaPT24-5B	TraesCS5B02G512000	5B	677537337	677539246	1	1614	537	11	OsPT8(87)	0	GGDYPLSATIMSE
TaPT25-5B	TraesCS5B02G512100	5B	677542981	677545165	-1	1509	503	10	OsPT8(87)	0	GGDYPLSATIMSE
TaPT26-5D	TraesCS5D02G472000	5D	512532864	512536289	1	1587	529	11	OsPT8(80)	0	GGDYPLSATIMSE
TaPT27-5D	TraesCS5D02G512900	5D	537087947	537089896	1	1410	470	9	OsPT8(88)	0	GGDYPLSATIMSE
TaPT28-5D	TraesCS5D02G513000	5D	537161285	537162607	-1	1410	470	9	OsPT8(87)	0	GGDYPLSATIMSE
TaPT29-6A	TraesCS6A02G223800	6A	420820498	420822475	-1	1602	533	12	OsPT8(64)	0	GGDYPLSATIISE
TaPht-myc	TraesCS6B02G257500	6B	462035116	462036717	1	1602	533	12	OsPT8(64)	0	GGDYPLSATIISE
TaPT30-6D	TraesCS6D02G211600	6D	298924441	298926287	1	1602	533	12	OsPT8(64)	0	GGDYPLSATIISE
TaPT31-7A	TraesCS7A02G070000	7A	35696602	35698417	1	1671	556	10	OsPT13(64)	0	GGDYPLSATIMSE
TaPT32-7D	TraesCS7D02G064200	7D	35551430	35553485	1	1668	555	9	OsPT13(64)	0	GGDYPLSATIMSE
TaPT33-7D	TraesCS7D02G064900	7D	36043328	36045147	-1	1671	556	10	OsPT13(63)	0	GGDYPLSATIMSE
TaPT34-Un	TraesCSU02G070800	Un	61731351	61732928	-1	1578	525	12	OsPT2(81)	0	GGDYPLSATIMSE

Table S2. Conserved motifs identified in the TaPHT1 family proteins.

Conserved motif sequences	Motif ID	Motif length	Site number
RRHGLHLLGTTSTWFLLDIAFYSQNLFQKDIFTAIGWIPPAKTMSALEEVYRIARAQALIALCGTVPGYWFTVAFIDIIGRFAIQLMGFFMMTVFMLGLA	Motif 1	113	50
HTPKGVMATLCFFRFWLGFGIGGDYPLSATIMSEYANKKTRGAFIAAVFAMQGFGIL	Motif 2	57	56
PZADYVWRIILMFGAIPALLTYYWRMKMPETARYTALVAKNAKQAASDMSKVLQVEI	Motif 3	57	56
VVLYALTFFFANFGPNSTTFIVPAEIFPARLRSTCHGISAAAGKAGAIVGAFGFLYA	Motif 4	57	56
ZQLNVLKALDVAKTQLYHFTAIVIAGMGFFTDAYDLFCISLVTKLLGRIYYTDPASN	Motif 5	57	55
PGTLPPNVSAAVNGVALCGTLAGQLFFGWLGDKLGRKSVYGMTLILMVJCSIASGLS	Motif 6	57	42
QDQKKPDAGYPPGIGVRNSLFVLAGTNFLGLLFTFLVPESK	Motif 7	41	39
GGIVTJIVSSAFRAAFPAPAYQDDAAAST	Motif 8	29	42
GKSLEELSGENEDDE	Motif 9	15	56
EEDVQGERAKGBEFGLFSRZF	Motif 10	21	54
LCGTVLGQLFFGWLGDRMGRKRIYGVTLKLMVVCSLASGLS	Motif 11	41	13
WPARRHACSSLFCHLHGAGSAMLYRVLDAVTSVKCETRRAR	Motif 12	41	6
HTSIRSSLLLLAGCNLVGVMFTLLLPESK	Motif 13	29	14
EPPGYSATVAEVEFIGRVEIQ	Motif 14	21	12
RRHGRDLLATSVCWFLLDVVFYSLNLFMKDIFS	Motif 15	33	6
GAVVLVLSARFKNTAAYETDQ	Motif 16	21	6
PPYSTYWQPHSSMN	Motif 17	14	7
LPGNVAGAVSG	Motif 18	11	7
DQARAAAVQPS	Motif 19	11	6
MARSEQ	Motif 20	6	6

Table S3. The parameters of selection pressure on duplicated gene pairs of the TaPHT1 family.

Duplication type	duplicated gene_1	duplicated gene_2	Ka	Ks	Ka_Ks	Divergence time (million years)
Tandem duplication	TaPT5-4A	TaPT6-4A	0.0016543	0.0229805	0.0719853	1.75
Tandem duplication	TaPT7-4A	TaPT8-4A	8.87E-04	0.0728183	0.0121781	5.55
Tandem duplication	TaPT7-4A	TaPT9-4A	0.0075741	0.1165856	0.0649663	8.89
Tandem duplication	TaPT7-4A	TaPT10-4A	0.0084646	0.1585604	0.0533841	12.09
Tandem duplication	TaPT8-4A	TaPT9-4A	0.0064181	0.0942791	0.0680752	7.19
Tandem duplication	TaPT8-4A	TaPT10-4A	0.0072733	0.1615909	0.0450106	12.32
Tandem duplication	TaPT9-4A	TaPT10-4A	0.0025426	0.1486327	0.0171063	11.33
Tandem duplication	TaPT13-4A	TaPT14-4A	0.0041867	0.0442741	0.0945641	3.37
Tandem duplication	TaPT16-4B	TaPT17-4B	0.0046928	0.1590135	0.0295123	12.12
Tandem duplication	TaPT16-4B	TaPT18-4B	0.0072723	0.1976424	0.0367951	15.06
Tandem duplication	TaPT17-4B	TaPT18-4B	0.0076412	0.1845701	0.0414001	14.07
Tandem duplication	TaPT20-4D	TaPT21-4D	0.003838	0.0275984	0.1390647	2.10
Tandem duplication	TaPT24-5B	TaPT25-5B	8.25E-04	0.0126425	0.0652894	0.96
Tandem duplication	TaPT27-5D	TaPT28-5D	0.0010059	0.0284694	0.0353315	2.17
Segmental duplication	TaPT1-2B	TaPT2-2D	0.0282761	0.0908912	0.3110978	6.93
Segmental duplication	TaPT4-4A	TaPT15-4B	0.009194	0.0992198	0.0926629	7.56
Segmental duplication	TaPT4-4A	TaPT19-4D	0.0056	0.0564852	0.0991415	4.31
Segmental duplication	TaPT15-4B	TaPT19-4D	0.0088107	0.0701506	0.1255965	5.35
Segmental duplication	TaPT5-4A	TaPT27-5D	0.0016538	0.0994298	0.0166329	7.58
Segmental duplication	TaPT8-4A	TaPT20-4D	0.001705	0.1411279	0.0120815	10.76
Segmental duplication	TaPT8-4A	TaPT16-4B	0.0025594	0.1991236	0.0128531	15.18
Segmental duplication	TaPT20-4D	TaPT16-4B	8.52E-04	0.1227793	0.0069385	9.36
Segmental duplication	TaPT12-4A	TaPT31-7A	0.0104127	0.1357074	0.0767288	10.34
Segmental duplication	TaPT14-4A	TaPT33-7D	0.0337799	0.277618	0.1216775	21.16
Segmental duplication	TaPT10-4A	TaPT17-4B	0.0059364	0.1944441	0.0305303	14.82
Segmental duplication	TaPT17-4B	TaPT10-4A	0.0059364	0.1944441	0.0305303	14.82
Segmental duplication	TaPT17-4B	TaPT21-4D	0.0033841	0.0697553	0.0485139	5.32
Segmental duplication	TaPT10-4A	TaPT21-4D	0.0025391	0.1811328	0.0140182	13.81
Segmental duplication	TaPT20-4D	TaPT10-4A	0.005554	0.1681628	0.0330275	12.82
Segmental duplication	TaPT10-4A	TaPT16-4B	0.006413	0.2180172	0.0294153	16.62
Segmental duplication	TaPT29-6A	TaPht-myc	0.0104714	0.0644144	0.1625626	4.91
Segmental duplication	TaPT29-6A	TaPT30-6D	0.004169	0.0414972	0.1004645	3.16
Segmental duplication	TaPht-myc	TaPT30-6D	0.0087812	0.0645276	0.1360844	4.92

Table S4. Primers names and sequences used in this study.

Name	Phytozome ID/ Genbank ID	Sequence (5'-3')	Primers purpose
TaPT1-2B-F	TraesCS2B02G059100.1	TCAGGCTGCTTGACATTGAC	q RT-PCR analysis
TaPT1-2B-R		GACGGCGAATCCATATTTGT	
TaPT2-2D-F	TraesCS2D02G045600.1	CTTATGCTAGTGTCTTGGTGC	
TaPT2-2D-R		AGGGACATATGTTGCCCTGC	
TaPT3-2D-F	TraesCS2D02G045700.1	AGCCAACACAGGCATTGA	
TaPT3-2D-R		GAAGAGGTCGTAGGCATCAGT	
TaPT4-4A-F	TraesCS4A02G301100.1	ATCGCCTTCTACAGCCAGA	
TaPT4-4A-R		TCACGAACACCTCTTTGAGC	
TaPT5-4A-F	TraesCS4A02G359900.1	GACGCTGATTCTCATGGTCA	
TaPT5-4A-R		CGTACTCGGACATGATGGTG	
TaPT6-4A-F	TraesCS4A02G360000.1	GTGGTTCCTGCTGGACATC	
TaPT6-4A-R		ATCCAGTTGATGCTCGTGAA	
TaPT7-4A-F	TraesCS4A02G416300.1	GCGATTATCCTCTCAGCGCC	
TaPT7-4A-R		GACGAGACGATGATTGTGACGATA	
TaPT8-4A-F	TraesCS4A02G416400.1	ATGTCGGAGTATGCTAAC	
TaPT8-4A-R		CAAATAGGATGCCAAACC	
TaPT9-4A-F	TraesCS4A02G416500.1	CTGGGTCTGCTCTTTTCGCT	
TaPT9-4A-R		AATGGAGTCATCGTCGCCAA	
TaPT10-4A-F	TraesCS4A02G416600.1	GAAAGCGCTGGACGTTGCC	
TaPT10-4A-R		GCAGGGTCGGTGTAGTAGATG	
TaPT11-4A-F	TraesCS4A02G417900.1	ATGTTCACTCTTGCCTTG	
TaPT11-4A-R		CGCTTTCTTCCTCCATTT	
TaPT12-4A-F	TraesCS4A02G418000.1	CGGAGAAATGGAGGAAGAAAG	
TaPT12-4A-R		ACGCTGTGGATGAACTCA	
TaPT13-4A-F	TraesCS4A02G418300.1	TGAAGATGCCTGAGACGGC	
TaPT13-4A-R		GAGGACGAACCAACACGTAGTC	
TaPT14-4A-F	TraesCS4A02G418400.1	GGTAAGTGTGGTGCCATCATT	
TaPT14-4A-R		AGGTGGTTCGCTTTCTTCC	
TaPT15-4B-F	TraesCS4B02G013200.1	ACATCTTCCCAGCCATCAAC	
TaPT15-4B-R		ACCCAGTAGCCTGGGAAAGT	
TaPT16-4B-F	TraesCS4B02G317000.1	GACATCATTGGGAGGTTT	
TaPT16-4B-R		CAAGTAGTCGTAAGGTATGG	
TaPT17-4B-F	TraesCS4B02G317100.1	ACACAAACAAGAGAGAACCAGA	
TaPT17-4B-R		GGGTCGGTGTAGTAGATGCG	

TaPT18-4B-F<br>TaPT18-4B-R	TraesCS4B02G317200.1	AACTGCACGGTACACAGCAC<br>GAGAAGAGGCCCCAGGTATC
TaPT19-4D-F<br>TaPT19-4D-R	TraesCS4D02G010900.1	CCGCCATTTTCCTCCAC<br>TCTCGGGCATCTTCATCC
TaPT20-4D-F<br>TaPT20-4D-R	TraesCS4D02G313700.1	CGCCTTCATCGACATTATTGGG<br>ACGCATACAGGAACCCGAAC
TaPT21-4D-F<br>TaPT21-4D-R	TraesCS4D02G313800.1	TCGGAATATGCTAACAAGA<br>CAAATAGGATGCCAAACC
TaPT22-5A-F<br>TaPT22-5A-R	TraesCS5A02G460300.1	CGGTTCGAGTCGCCAGTAAA<br>GAGGGAACGCTGGGATCAAA
TaPT23-5B-F<br>TaPT23-5B-R	TraesCS5B02G470100.1	ACCGACGCCTACGACCT<br>GCCAGCCGAAGAAGAGC
TaPT24-5B-F<br>TaPT24-5B-R	TraesCS5B02G512000.1	CATCATCCTCATGCTCGGCG<br>GAGCTCGTCCATCTTCTCCG
TaPT25-5B-F<br>TaPT25-5B-R	TraesCS5B02G512100.1	CTACACCGACCTCTCCAAGC<br>GATGGAGCAGATGACCATGA
TaPT26-5D-F<br>TaPT26-5D-R	TraesCS5D02G472000.1	CTTCACGAGCATCAACTGGA<br>ACGACGTCGATGAGGAAGAC
TaPT27-5D-F<br>TaPT27-5D-R	TraesCS5D02G512900.1	CACCATCATGTCCGAGTACG<br>AAGGCGGATGAGATGATGAG
TaPT28-5D-F<br>TaPT28-5D-R	TraesCS5D02G513000.1	GACGCTCATTCTCATGGTCA<br>CGTACTCGGACATGATGGTG
TaPT29-6A-F<br>TaPT29-6A-R	TraesCS6A02G223800.1	ACGTACGCGGAAGACCCCAT<br>TGTCCTGCTCGCCGCTGAAC
TaPht-myc-F<br>TaPht-myc-R	TraesCS6B02G257500.1	GCGTATGACGAAGACCACAT<br>TGTCCTGCTCGCCGCTGAGT
TaPT30-6D-F<br>TaPT30-6D-R	TraesCS6D02G211600.1	GCGTACGCAGAAGACCACAT<br>TGTCCTGCTCGCCGCTGAAT
TaPT31-7A-F<br>TaPT31-7A-R	TraesCS7A02G070000.1	AGACCTACAAGATAGCCCGC<br>AGGATGAAAGTGGTGGAGTTG
TaPT32-7D-F<br>TaPT32-7D-R	TraesCS7D02G064200.1	GGAACAGCTGTCGTCGTCTT<br>AATTGGCAGCTTTGCTGACG
TaPT33-7D-F<br>TaPT33-7D-R	TraesCS7D02G064900.1	TAAGTGTGGTGCCATCAT<br>CTGCCAGAAGAAGGAGAG
TaPT34-Un-F<br>TaPT34-Un-R	TraesCSU02G070800.1	CAAATAGGATGCCAAACC<br>GGAATATGCTAACAAGAAGA
TaActin-F<br>TaActin-R	KC775780.1	CCAGGTATCGCTGACCGTAT<br>GCTGAGTGAGGCTAGGATGG

TaPR4a-F<br>TaPR4a-R	AJ006098.1	CGTCTTCACCAAGATCGACA<br>GGCAGTCGACGAACTGGTA	
TaPR4b-F<br>TaPR4b-R	AJ006099.1	CTTCACCAAGATCGACACCA<br>AGCAAGCTAGCCTTTGATCG	
TaPR2-F<br>TaPR2-R	DQ090946.1	CCGGCCATACTACCCGGC<br>ACACCTTGATGGCGCTGAGA	
TaPR10-F<br>TaPR10-R	CV778999.1	ACGGAGCGGATGTGGAAG<br>GCCACCTGCGACTTGAGC	
TaPT29-6A-F1<br>TaPT29-6A-R1	TraesCS6A02G223800.1	GGAATTCATGGCACGGCAGCAGC<br>CGGTCGACCTACAAGGGAAGAACCT	Subcellular location vector construct
TaPT29-6A-F2<br>TaPT29-6A-R2	TraesCS6A02G223800.1	GTGAGTAAGGTTACCGAATCAGCGGCGAGCAGG<br>CGTGAGCTCGGTACCGGA ACTTGATGTCCCTGA	VIGS vector construct

Table 5. Promoter elements in each *TaPHT1* gene.

Gene name	P1BS (GNATATNC)	OSEROOTNODULE (AAAGAT)	NODCON2GM (CTCTT)	Root motif box (ATATT/AATAT)	W-box (TTGACY)
TaPT1-2B	-1098	-875	-1489, -1454	-1497, -1034, -882, -736, -711, -504	-1653
TaPT2-2D	-810	-564	-175	-852, -750, -508, -385, -277, -48	_
TaPT3-2D	_	_	-512	-595, -280, -154	_
TaPT4-4A	-605, -318	_	-1640	-1223, -1211, -913	-976, -798, -679
TaPT5-4A	_	_	-1874, -1733, -1136, -539	_	
TaPT6-4A	-1273	-1644	_	-1874, -1851, -1694, -1603, -1468, -1406, -1357, -1325, -1272, -353, -294, -162	-1299
TaPT7-4A	-1556, -640, -422	-44	-1692, -598	-1811, -1687, -1554, -1538, -1451, -855, -248, -140	-1997, -1965, -474
TaPT8-4A	-447, -126	_	-1952, -1548, -1531, -1439	-973, -461	-1527, -1333
TaPT9-4A	-633, -385	_	-1413, -1330, -1148, -875	-1936, -1852, -1805, -668, -401, -203, -	-1031, -437, -215

				137, -113	
TaPT10-4A	-1541, -675	_	-1942, -1921, -288	-1901, -1691, -1523, -1270, -1142, -945, -798, -451, -240, -173, -130	-728, -252
TaPT11-4A	_	_	-452	-649, -557, -118	-1965, -1713, -1228
TaPT12-4A	_	_	-1720	-1972, -842, -732, -690, -445	-1628
TaPT13-4A	-1155	-992	-1582, -32	-1119, -877, -218	_
TaPT14-4A	-1542	-1348, -914	-1233	-687, -128	-1606
TaPT15-4B	-514, -225	_	_	-931, -513, -224	-715, -596
TaPT16-4B	-1623, -644, -423	-44	-1358, -602	-1827, -1564, -1477, -1290, -1149, -248	-475
TaPT17-4B	-1703, -1464	_	-593	-1791, -1584, -1462, -1083, -829, -141	-1516, -1295, -1252, -838, -651
TaPT18-4B	-1621, -391	_	-945	-1751, -1603, -1550, -1365, -1233, -674, -208, -142	-1856, -443, -220
TaPT19-4D	-600, -316	_	-1587	-1202, -1190, -913	-1117, -1102, -798
TaPT20-4D	-1881, -997	_	-1342, -1201, -613	-1592	-1933, -1713, -855
TaPT21-4D	-1458, -399	_	_	-1831, -1600, -1587, -1440, -1387, -1199, -1067, -676, -415, -218, -152, -124	-1688, -451, -230, -198
TaPT22-5A	-1088, -346	_	-917, -537	-1729, -879, -676, -657, -491	-1619, -1090, -1123
TaPT23-5B	-1422, -316	_	-1039, -712, -479, -404, -69	-1726, -661, -627	-1527, -1457, -1267
TaPT24-5B	_	-1708	-1894, -1362	-1684, -1664, -842, -730, -632, -329, -290, -163, -157	-1837
TaPT25-5B	_	-1708	-1894, -1362	-1663, -841, -631, -328, -290, -156	-362

TaPT26-5D	-1568	_	-1455, -484	-1863,-1006, -713, -684, -306	-1676, -1603, -1401
TaPT27-5D	-1863	-1873	-1550	-1580, -1237, -411, -261	-1512, -1499, -1494
TaPT28-5D	_	-645	-1116, -1065	-1700, -714, -590, -566, -286	-588
TaPT29-6A	-1722, -696	-1016	_	-1047, -694, -622	-595
TaPht-myc	-218	-1843	-1257	-1212, -752, -653	-975, -626, -427
TaPT30-6D	_	-1506, -410	-1289	-1843, -1537, -1265, -697, -675	-1395, -1013, -809, -598
TaPT31-7A	-1375	-1158	-431	-824, -118	-1870, -1555, -1225, -1119
TaPT32-7D	-1073	-1669, -879	_	-1913, -1830, -1772, -1685, -1678, -1657, -1030, -1014, -364	_
TaPT33-7D	_	_	-1580, -732, -542, -97	-600, -199, -92	-1203, -1033, -729
TaPT34-Un	-1874, -997	_	-1201, -613	-1700, -1592	-1926, -1712, -855

```
TaPT29-6A   MARQQLQVLHALDVARTQRYHAWAVVIAGMGFFADAYDIFCITLVTKLLGRIYYQVPGQREPGMLPRRIEAAINGVTFCGMIVGQLLFGWLGDKVGRKKF   100
TaPT30-6D   MARQQLQVLHALDVARTQRYHAWAVVIAGMGFFADAYDIFCITLVTKLLGRIYYQVPGQREPGMLPRRIEAAINGVTFCGMIVGQLLFGWLGDKVGRKMF   100
TaPht-myc   MARQQLQVLHALDVARTQRYHAWAVVIAGMGFFADAYDIFCITLVTKLLGRIYYHVPGQCVPGMLPRRIEAAINGVTFCGMIVGQLLFGWLGDKVGRKMF   100
Consensus   marqqlqvlhaldvartqryhawavviagmgffadaydifcitlvtkllgriyy vpgq  pgmlprrieaaingvtfcgmivgqllfgwlgdkvgrk f

TaPT29-6A   YGKTIMLMIMGSFLSGLSFGNTADGVMATLCFFRFWLGVGIGGDYPLSATIISEYSNKRTRGSLIAAVFAMEGFGILAGCIVTLVVSATFQARFDPPTYA   200
TaPT30-6D   YGKTIMLMIMGSFLSGLSFGNTADGVMATLCFFRFWLGVGIGGDYPLSATIISEYSNKRTRGSLIAAVFAMEGFGILAGCIVTLVVSATFQARFNPPAYA   200
TaPht-myc   YGKTIMLMIMGSFLSGLSFGNTADGVMATLCFFRFWLGVGIGGDYPLSATIISEYSNKRTRGSLIAAVFAMEGFGILAGCIVTLVVSATFQARFNPPAYD   200
Consensus   ygktimlmimgsflsglsfgntadgvmatlcffrfwlgvgiggdyplsatiiseysnkrtrgsliaavfamegfgilagcivtlvvsatfqarf pp y

TaPT29-6A   EDPMASVPPQADYVWRIILMVGAIPAVFTYRWRVMMPETARYTALVARDAEKAARDMSKVLKVEFSGEQDKIEGFTKDRDYGVFSRRFARRHGWHLVGAV   300
TaPT30-6D   EDHMASVPPQADYVWRIILMVGAIPAVFTYRWRVMMPETARYTALVARDAEKAARDMSKVLKVEFSGEQDKIEGFTKDRDYGVFSRRFARRHGWHLVGAV   300
TaPht-myc   EDHMASVPPQADYVWRIILMVGAIPAVFTYRWRVMMPETARYTALVARDAEKAARDMSKVLKVELSGEQDKIEGFTKDRDYGVFSRRFARRHGWHLVGAV   300
Consensus   ed masvppqadyvwriilmvgaipavftyrwrvmmpetarytalvardaekaardmskvlkve sgeqdkiegftkdrdygvfsrrfarrhgwhlvgav

TaPT29-6A   ASWFVLDIVFYSQIILQEEIFRDIKWIPEANSMSALEEAYRVARAQAIIALCGTLPGYWFTIAFVDVVGRKAIQFLGFTMMKGLMLVVAGFYHQLTQPGR   400
TaPT30-6D   ASWFVLDIVFYSQIILQEEIFRDIKWIPEANSMSALEEAYRVARAQAIIALCGTLPGYWFTIAFVDVVGRKAIQFLGFTMMKGLMLVVAGFYHQLTQPGR   400
TaPht-myc   ASWFVLDIVFYSQIILQEEIFRDIKWIPEANSMSALEEAYRVARAQAIIALCGTLPGYWFTIAFVDVVGRKAIQFLGFTMMKGLMLVVAGLYHQLTQPGR   400
Consensus   aswfvldivfysqiilqeeifrdikwipeansmsaleeayrvaraqaiialcgtlpgywftiafvdvvgrkaiqflgftmmkglmlvvag yhqltqpgr

TaPT29-6A   RIWLVVMYAFTFFFANFGPNSTTFIIPAEIFPAHVRTTCHGISSAAGKVGAIVGTFGFLYASQRADGSNETETGYPSGIGVRASLFVLAACNVLGIIFTC   500
TaPT30-6D   RIWLVVMYAFTFFFANFGPNSITFIIPAEIFPAHVRTTCHGISSAAGKVGAIVGTFGFLYASQRADGSNETETGYPSGIGVRASLFVLAACNVLGIIFTC   500
TaPht-myc   RIWLVVMYAFTFFFANFGPNSTTFIIPAEVFPAHVRTTCHGISSAAGKVGAIVGTFGFLYASQRADGSNERETGYPSGIGVRASLFVLAACNVLGIIFTC   500
Consensus   riwlvvmyaftfffanfgpns tfiipae fpahvrttchgissaagkvgaivgtfgflyasqradgsne etgypsgigvraslfvlaacnvlgiiftc

TaPT29-6A   LLPEPNGRSLEEVSGEPINGEDADLGDSKVLP   532
TaPT30-6D   LLPEPNGRSLEEVSGEPINGEDADLGDSKVLP   532
TaPht-myc   LLPEPNGRSLEEVSGEPINGEDADLGDSKVLP   532
Consensus   llpepngrsleevsgepingedadlgdskvlp
```

Figure S1. Alignment of TaPT29-6A, TaPT30-6D and TaPht-myc protein sequences using DNAman software. The sequence used to construct the TRV: *TaPT29-6A* vector is shown in the rectangle.

Motif 1

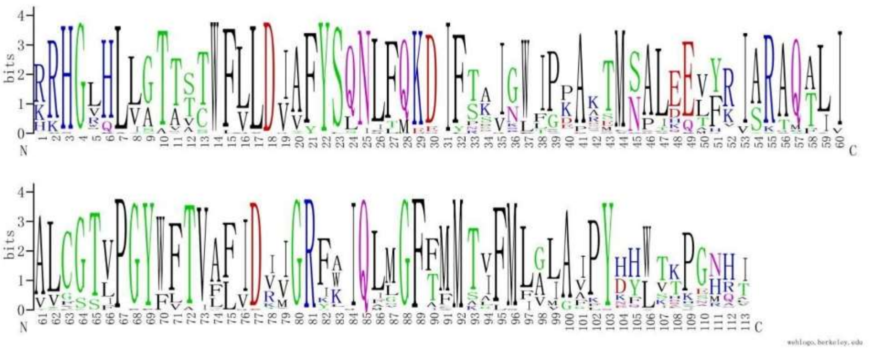

Motif 2

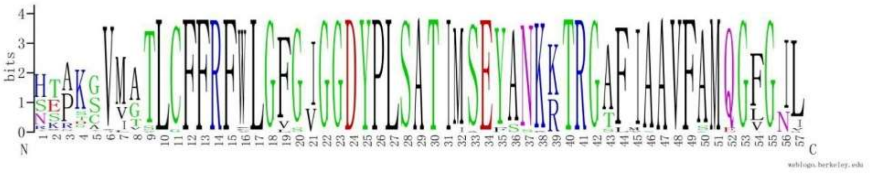

Motif 3

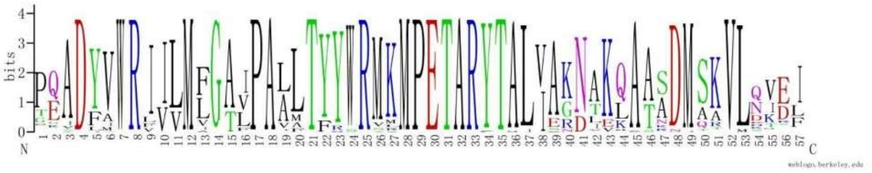

Motif 4

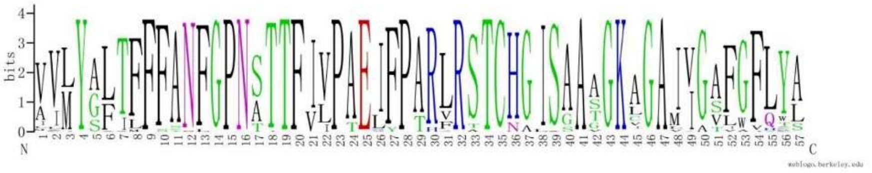

Motif 5

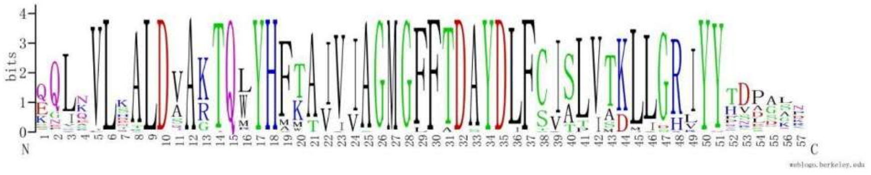

Motif 6

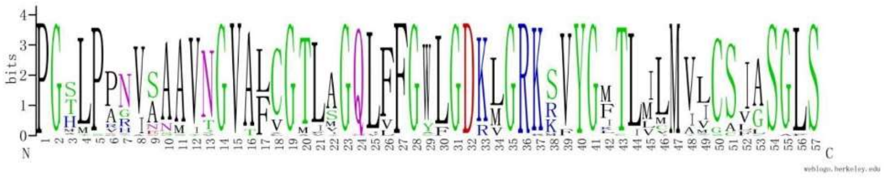

Motif 7

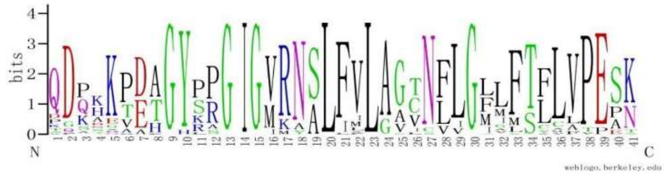

Motif 8

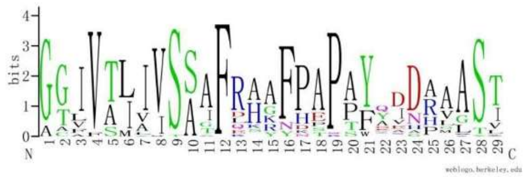

Motif 9

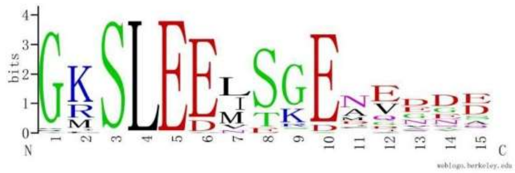

Motif 10

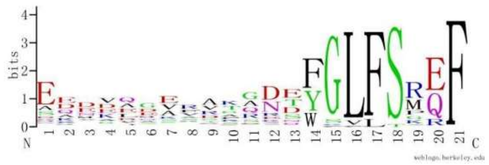

Motif 11

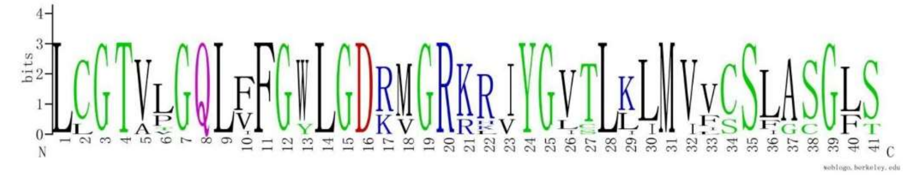

Motif 12

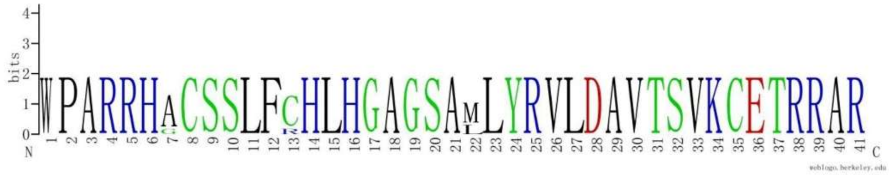

Motif 13

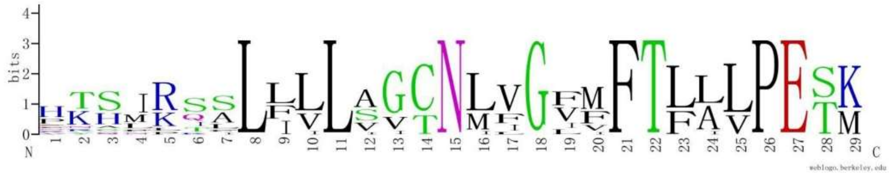

Motif 14

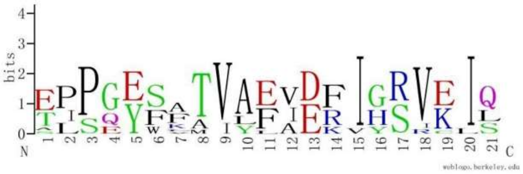

Motif 15

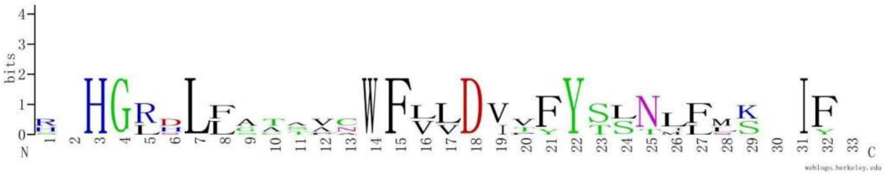

Motif 16

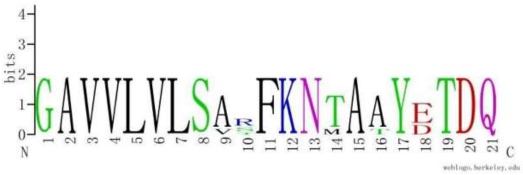

Motif 17

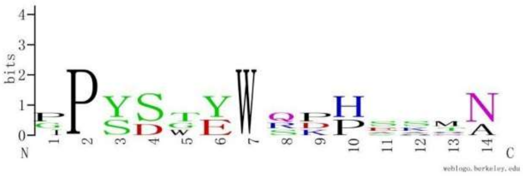

Motif 18

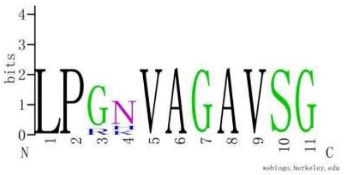

Motif 19

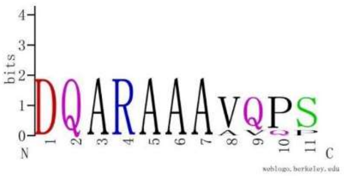

Motif 20

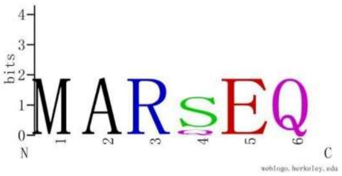

Figure S2. Twenty conserved motifs of a set of 57 PHT1 protein sequences identified by MEME and visualized using Weblogo. The parameters were as follows: motif count, 20; motif sites, 6 to 200; motif

width, 6 to 200; site distribution, zero or one occurrence per sequence; discovery mode, optimal E-value of the motif information content.

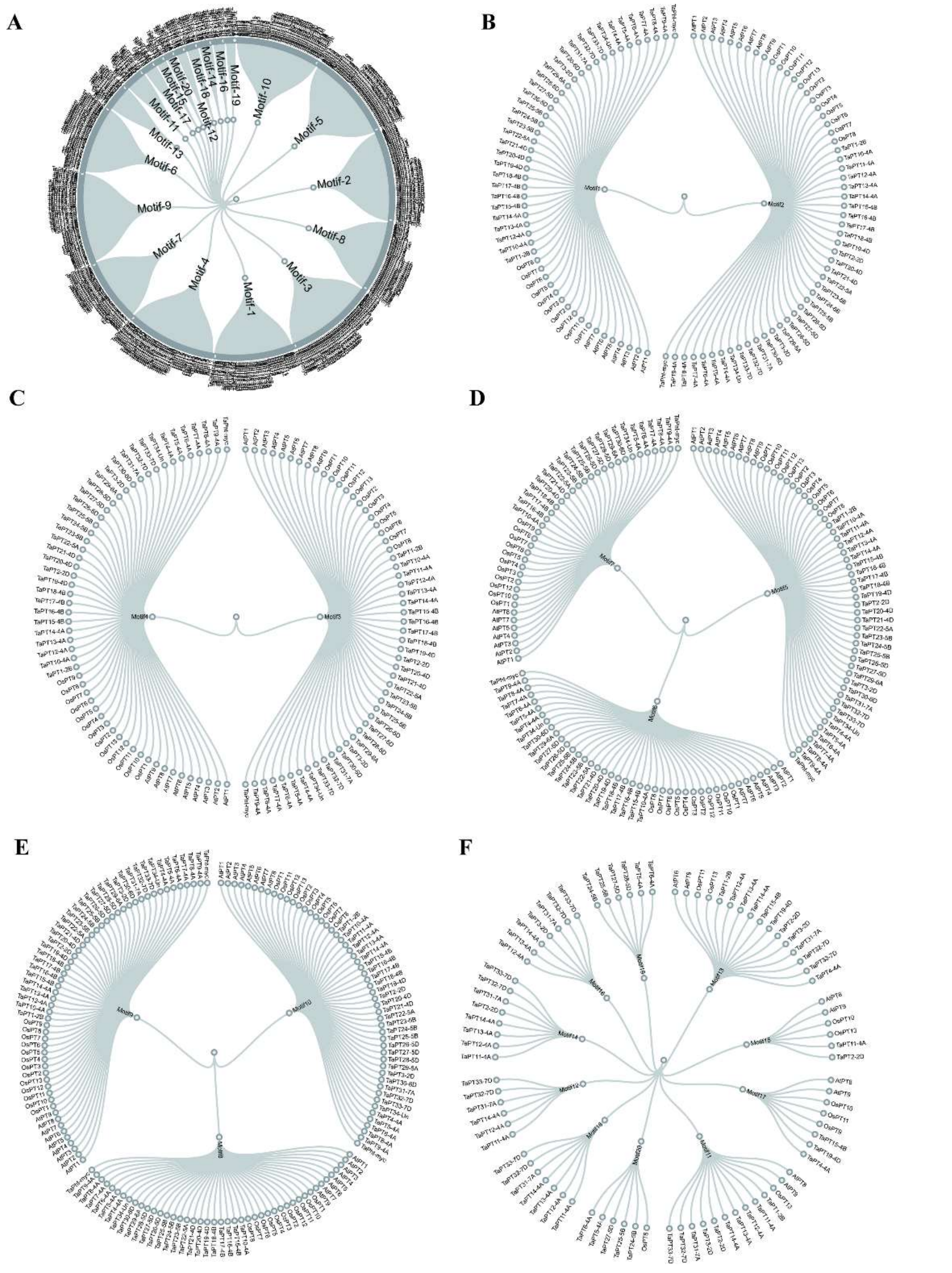

Figure S3. Clustering diagram of the PHT1 family in *Arabidopsis*, rice and wheat based on the presence or absence of motifs. (A) Clustering of all PHT1 members based on all the motifs. (B) Clustering of PHT1 proteins containing motifs 1 and 2. (C) Clustering of PHT1 proteins containing motifs 3 and 4. (D) Clustering of PHT1 proteins containing motifs 5, 6 and 7. (E) Clustering of PHT1 proteins containing motifs 8, 9 and 10 were clustered. (F) Clustering of PHT1 proteins containing motifs 11-20.

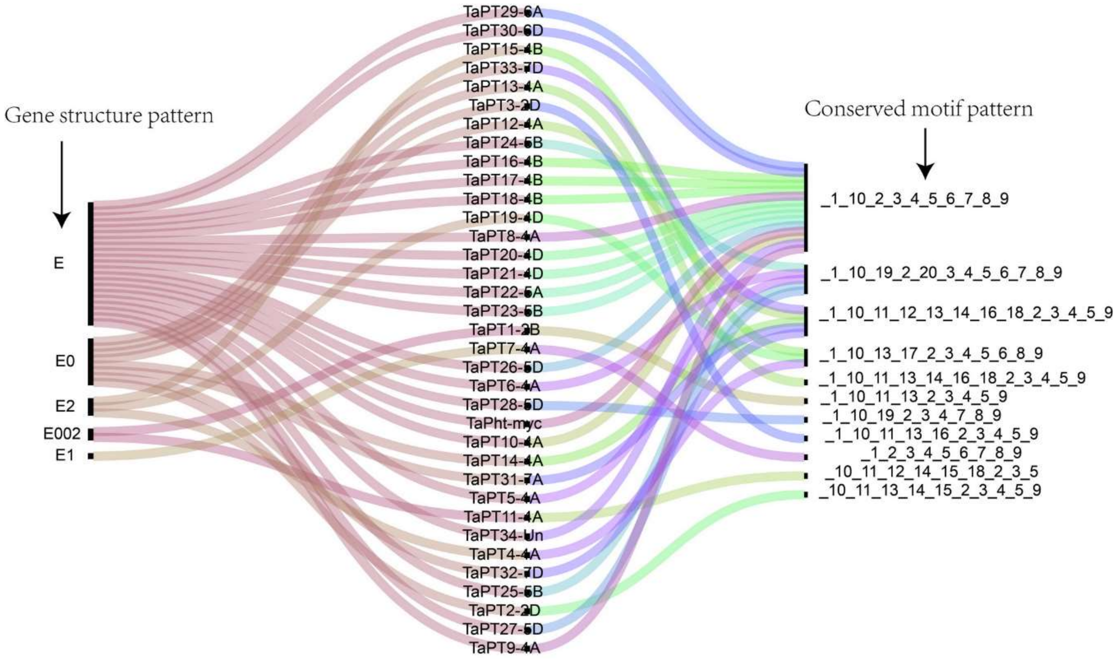

Figure S4. Alluvial diagram of correlations between gene structures and motif architectures in members of the PHT1 family in wheat.

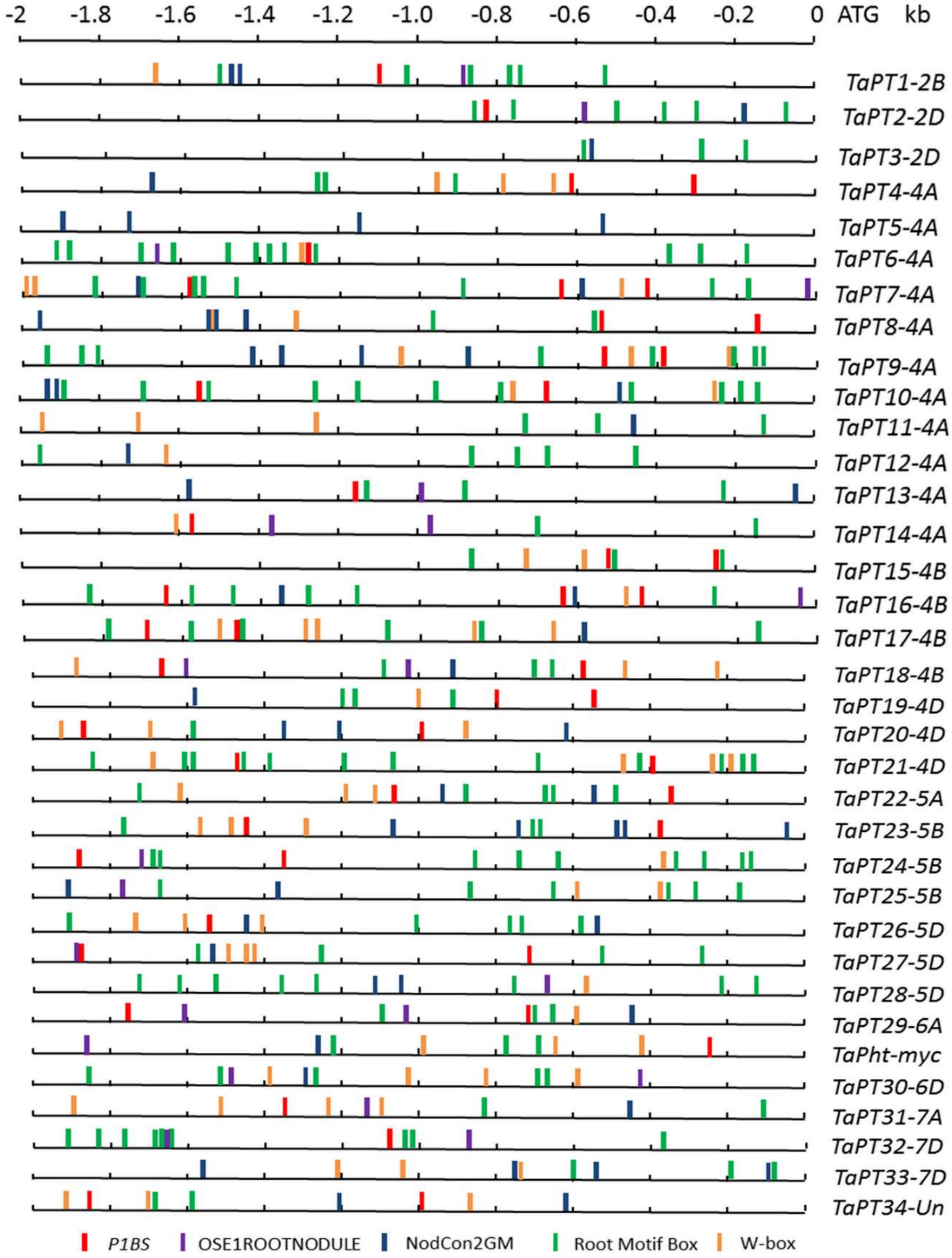

Figure S5. Putative *cis*-regulatory elements involved in Pi and AM responses in the promoter sequences of the 35 *TaPHT1* genes.

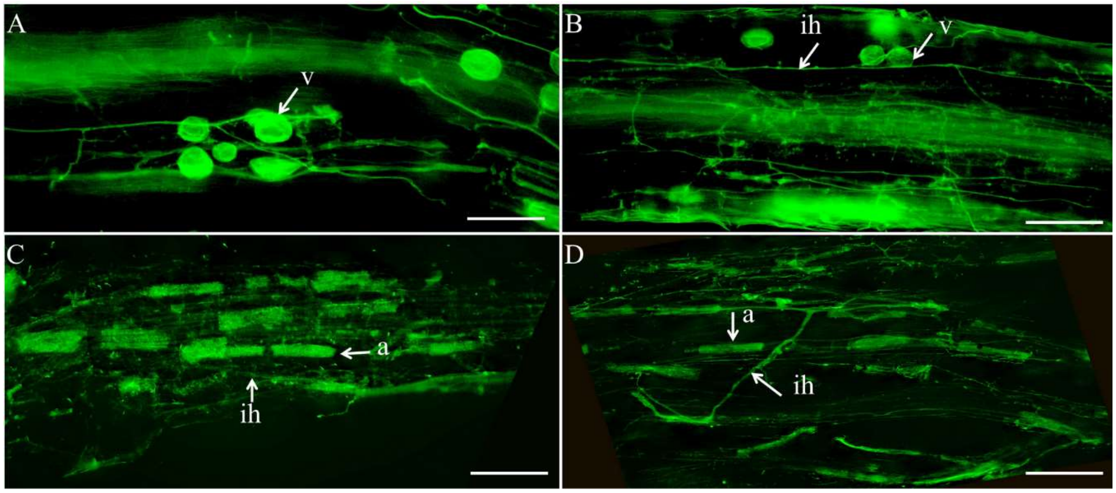

Figure S6. AM symbiotic wheat roots stained with WGA-AlexaFluor 488 to reveal fungal structures, including: internal hyphae (ih), vesicles (v) and arbuscules (a). Wheat roots were colonized with *G. versiforme* (A and C) and *F. mosseae* (B and D). Bar=20 μm.

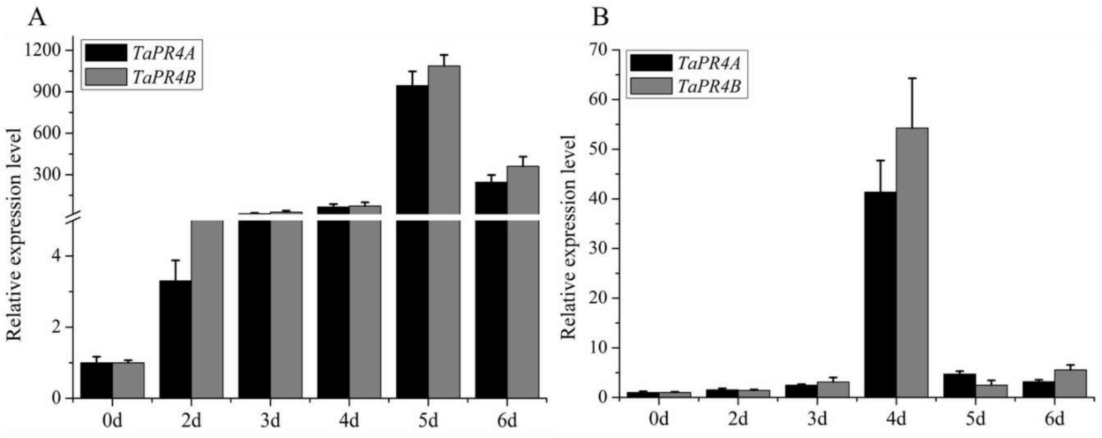

Figure S7. Relative expression levels of *TaPR4A* and *TaPR4B* marker genes in *Ggt* (A) and *B. sorokiniana* (B) infected wheat roots at indicated time-points. Bars represent means of three biological replications with standard errors.
